# Supplementary material for: Delineating the Biosynthesis of Gentamicin X2, the Common Precursor of the Gentamicin C Antibiotic Complex
Source: Chem Biol. 2015 Feb 19;22(2):251–61. doi: 10.1016/j.chembiol.2014.12.012 (PMC4340712; doi:10.1016/j.chembiol.2014.12.012)
Supplement: Document S1. Table S1, Figures S1–S5, and Supplemental Experimental Procedures [file mmc1.pdf]

Chemistry & Biology, Volume *22*

Supplemental Information

Delineating the Biosynthesis of Gentamicin X2, the Common Precursor of the Gentamicin C Antibiotic Complex

Chuan Huang, Fanglu Huang, Eileen Moison, Junhong Guo, Xinyun Jian, Xiaobo Duan, Zixin Deng, Peter F. Leadlay, and Yuhui Sun

Supplemental Information

<u>Inventory of Supplemental Information</u>

Table S1. LC-ESI-HRMS analysis of *genD2-genS2-genN-genD1* knock-out mutants. Related to Figure 2.

Figure S1. In-frame deletion of *genD2*, *genS2*, *genN* and *genD1* in *M. echinospora* ATCC 15835, and complementation of ΔgenD2, ΔgenS2, ΔgenN, ΔgenD1, ΔgenD2ΔgenK and ΔgenS2ΔgenK. Related to Figure 2.

Figure S2. MS and MS/MS spectra of gentamicin-related intermediates isolated from mutants of *M. echinospora*. Related to Figure 2.

Figure S3. LC-ESI-HRMS analysis of the production of the gentamicin C complex and gentamicin-relate intermediates by *Micromonospora echinospora* mutants fed with gentamicin A or G418. Related to Figure 2.

Figure S4. Characterization of purified recombinant GenD2, GenS2, GenN and GenD1. Related to Figures 3 and 4.

Figure S5. LC-ESI-HRMS analysis of GenD2-, GenS2- and GenN-catalyzed modifications on kanamycin B and tobramycin. Related to Figure 3.

Supplemental Experimental Procedures

1. List of oligonucleotide primers used in this study
2. List of bacterial strains and plasmids used in this study
3. Structures of 3''-dehydro-3''-amino-gentamicin A2 and its homologs
4. Purification of gentamicin A2
5. Kinetic study of GenN with kanamycin B
6. Conditions for LC-ESI-MS analyses

Table S1. LC-HRMS analysis of *genD2-genS2-genN-genD1* knock-out mutants

Strain	**Gentamicin C complex production** ($\times 10^5$)					**Intermediates production** ($\times 10^5$				
	**C1a**	**C2b**	**C2**	**C2a**	**C1**	**A2**	**A**	**A2e**	**X2**	**DAA2**
wild-type	12.6	1.3	59.8	10.0	95.9	2.1	3.4	ND	2.2	ND
ΔgenD2	ND	ND	ND	ND	ND	25.6	ND	30.3	ND	ND
ΔgenS2	ND	ND	ND	ND	ND	47.4	ND	46.5	ND	ND
ΔgenD2ΔgenK	ND	ND	ND	ND	ND	69.1	ND	ND	ND	ND
ΔgenS2ΔgenK	ND	ND	ND	ND	ND	70.6	ND	ND	ND	ND
ΔgenD2ΔgenK::*genK*	ND	ND	ND	ND	ND	27.8	ND	42.3	ND	ND
ΔgenS2ΔgenK::*genK*	ND	ND	ND	ND	ND	27.8	ND	36.8	ND	ND
ΔgenD1	ND	ND	ND	ND	ND	13.7	19.7	5.6	ND	ND
ΔgenN	ND	ND	ND	ND	ND	19.1	ND	18.1	ND	0.7

ND, not detected

Figure S1. In-frame deletion of *genD2*, *genS2*, *genN* and *genD1* in *M. echinospora* ATCC 15835, and complementation of ΔgenD2, ΔgenS2, ΔgenN, ΔgenD1, ΔgenD2ΔgenK and ΔgenS2ΔgenK. Related to Figure 2.

Schematic representation of the in-frame deletions and complementations, and Southern blot confirmations are shown for each mutant. The arrows indicate the expected size of the fragments from the wild-type and mutants chromosomal DNA, respectively.

A. ΔgenD2

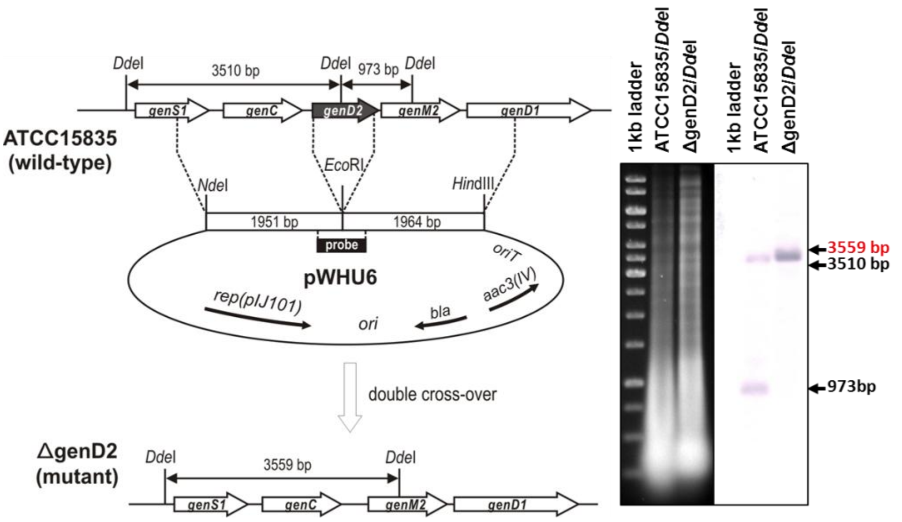

B. ΔgenS2

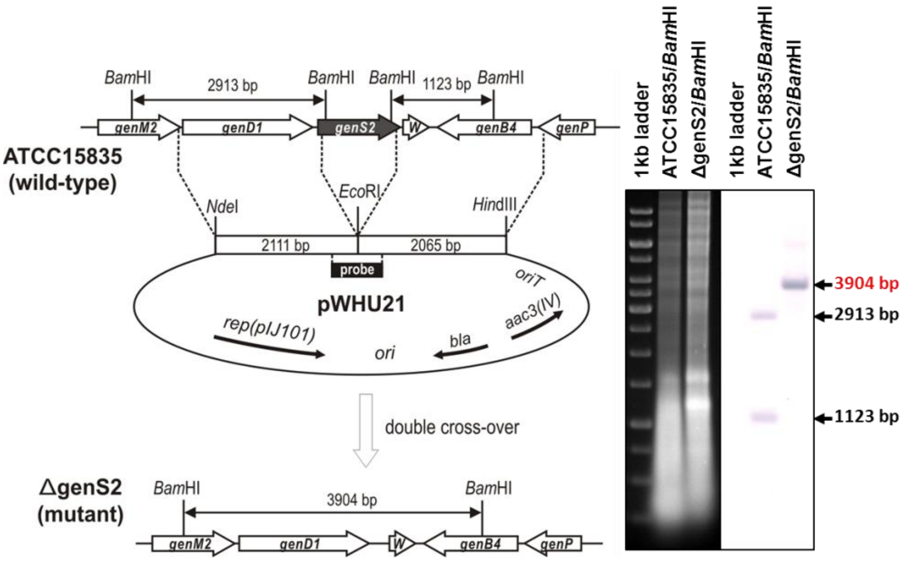

C. ΔgenD2ΔgenK

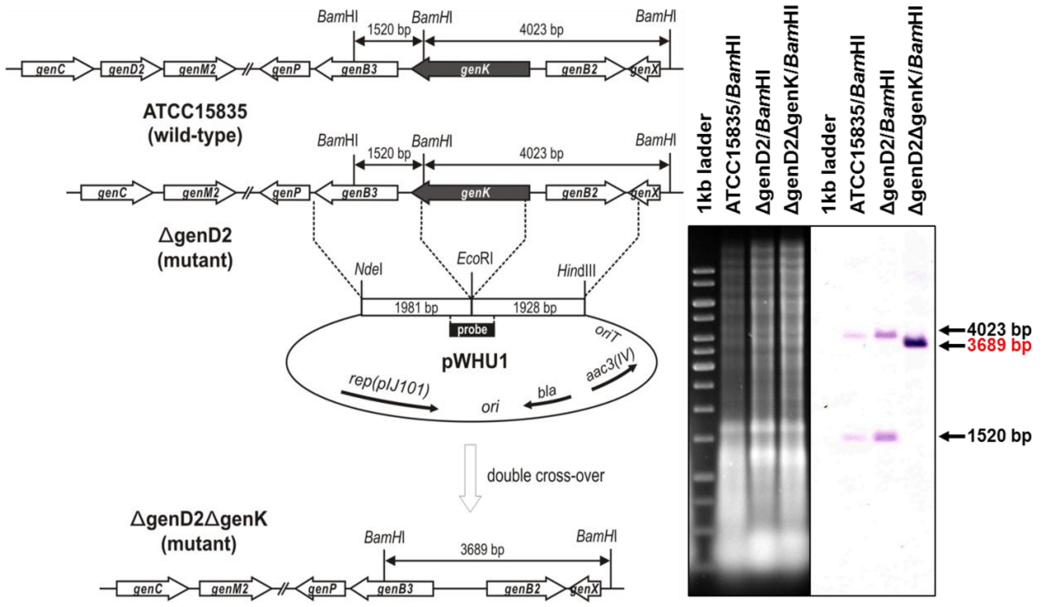

D. ΔgenS2ΔgenK

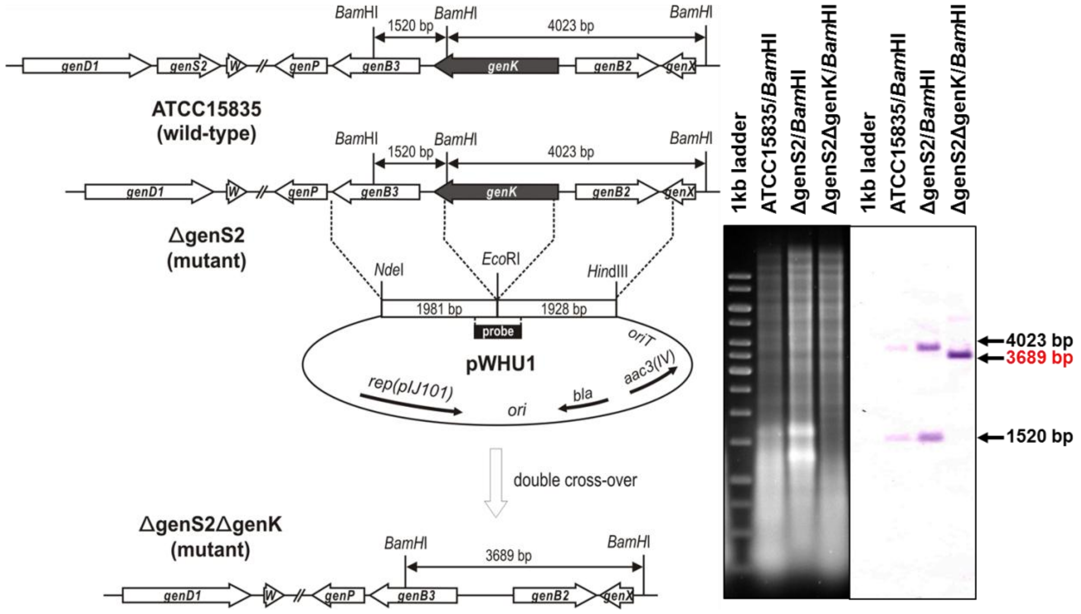

E. ΔgenN

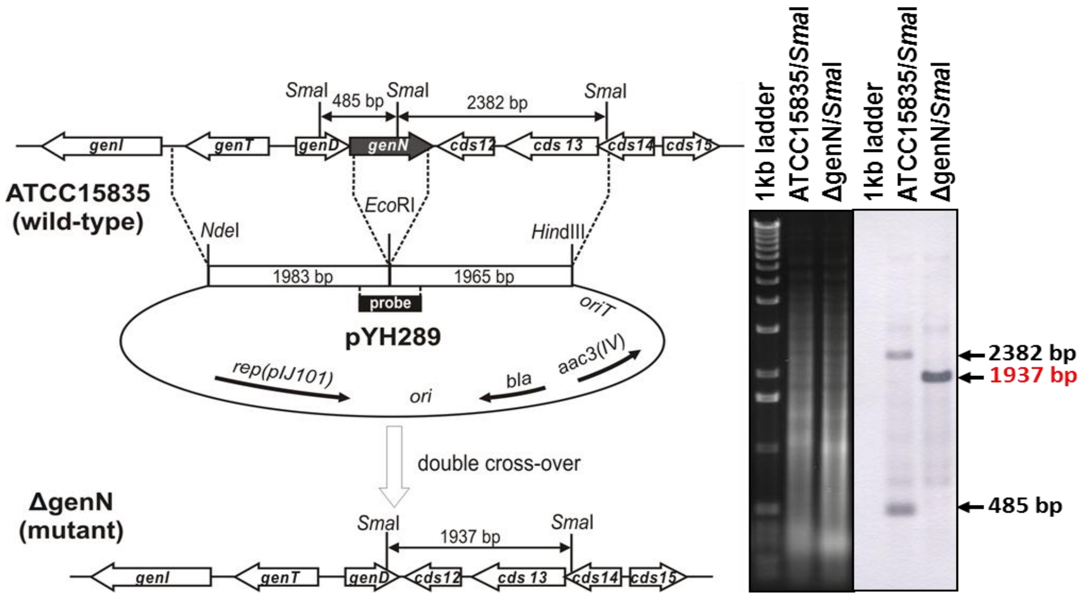

F. ΔgenD1

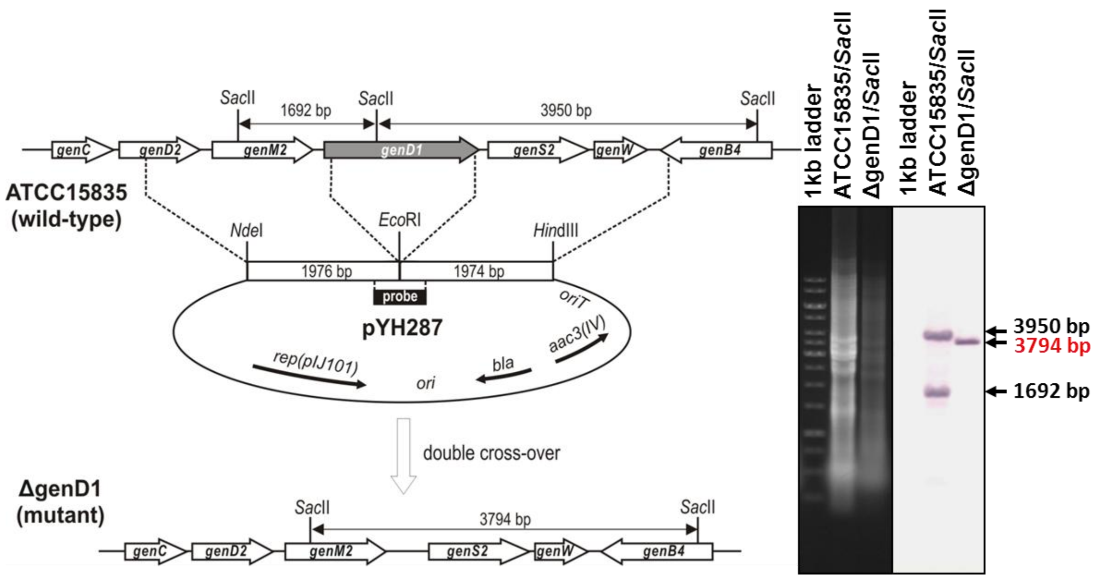

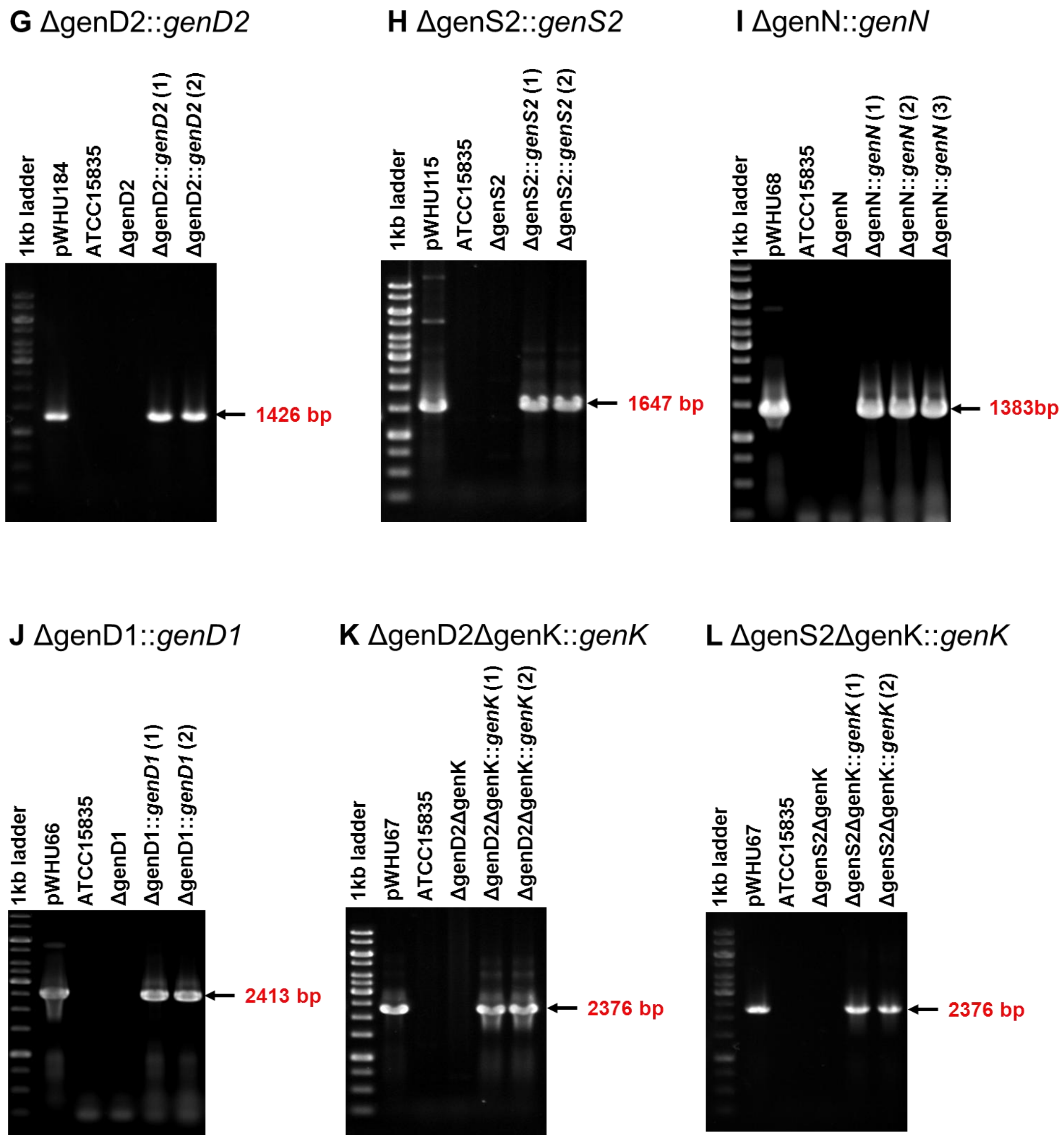

G ΔgenD2::genD2
1kb ladder
pWHU184
ATCC15835
ΔgenD2
ΔgenD2::genD2 (1)
ΔgenD2::genD2 (2)
1426 bp
H ΔgenS2::genS2
1kb ladder
pWHU115
ATCC15835
ΔgenS2
ΔgenS2::genS2 (1)
ΔgenS2::genS2 (2)
1647 bp
I ΔgenN::genN
1kb ladder
pWHU68
ATCC15835
ΔgenN
ΔgenN::genN (1)
ΔgenN::genN (2)
ΔgenN::genN (3)
1383bp
J ΔgenD1::genD1
1kb ladder
pWHU66
ATCC15835
ΔgenD1
ΔgenD1::genD1 (1)
ΔgenD1::genD1 (2)
2413 bp
K ΔgenD2ΔgenK::genK
1kb ladder
pWHU67
ATCC15835
ΔgenD2ΔgenK
ΔgenD2ΔgenK::genK (1)
ΔgenD2ΔgenK::genK (2)
2376 bp
L ΔgenS2ΔgenK::genK
1kb ladder
pWHU67
ATCC15835
ΔgenS2ΔgenK
ΔgenS2ΔgenK::genK (1)
ΔgenS2ΔgenK::genK (2)
2376 bp

Figure S2. MS and MS/MS spectra of gentamicin-related intermediates isolated from mutants of *M. echinospora*. Related to Figure 2.

Selective ion monitoring on (A) $[M+H]^+$(m/z 456.2188) ion of gentamicin A2 (**3**); (B) $[M+H]^+$(m/z 470.2344) ion of gentamicin A2e (**7**); (C) $[M+H]^+$(m/z 455.23478) ion of 3''-dehydro-3''-amino-gentamicin A2 (DAA2, [**9**]); (D) $[M+H]^+$(m/z 469.2504) ion of gentamicin A (**6**); (E) $[M+H]^+$(m/z 483.2661) ion of gentamicin Ae (**10**)

A

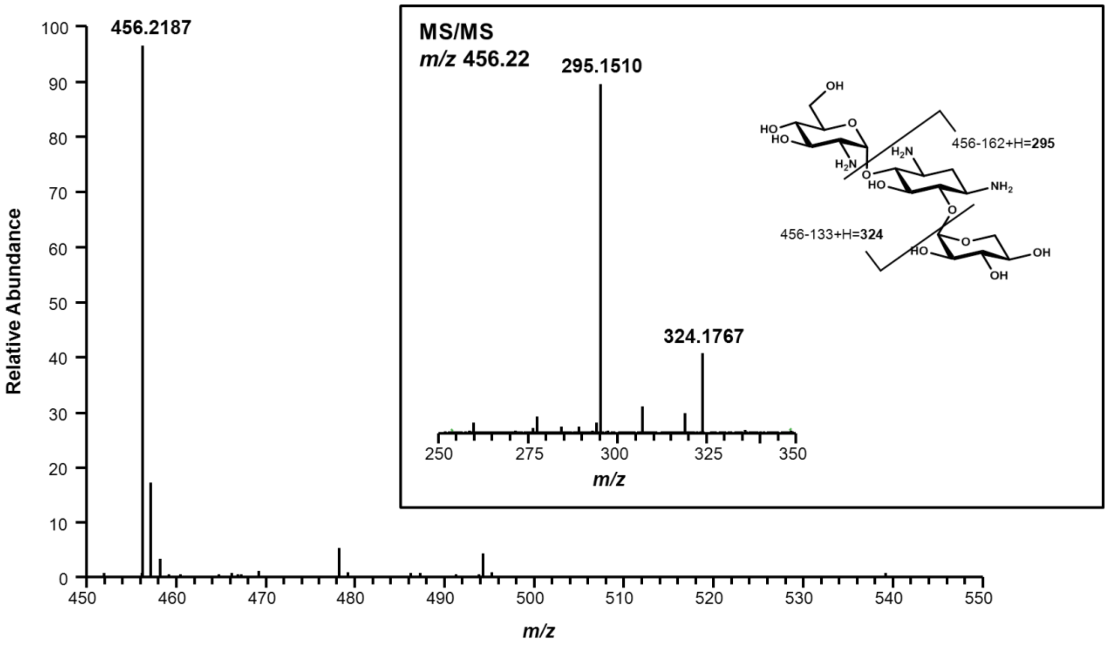

B

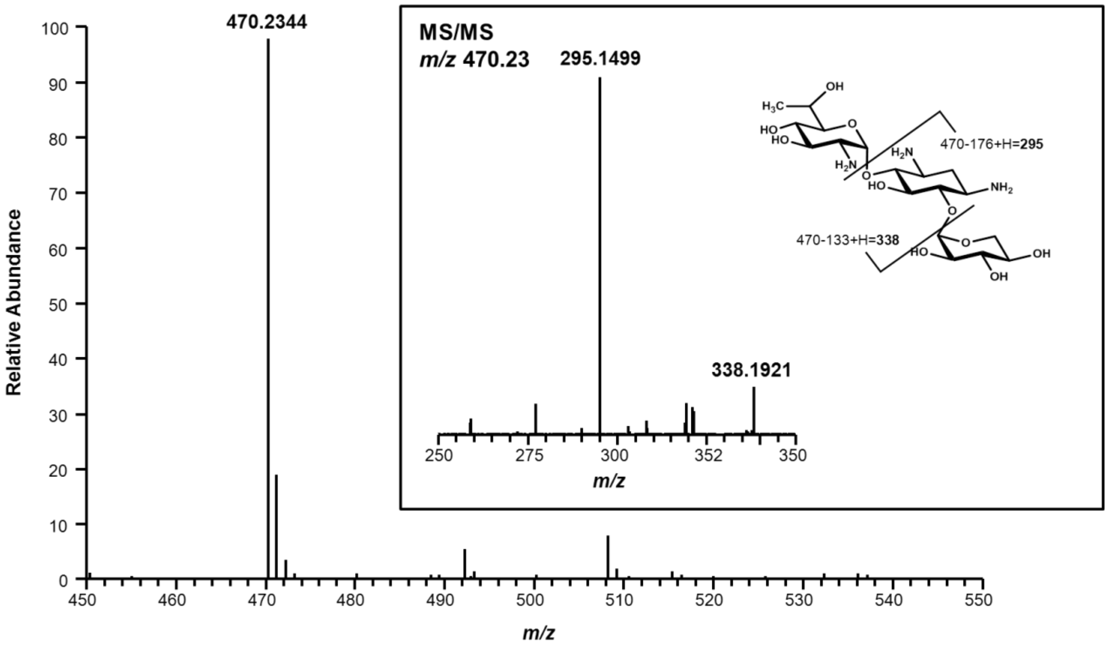

C

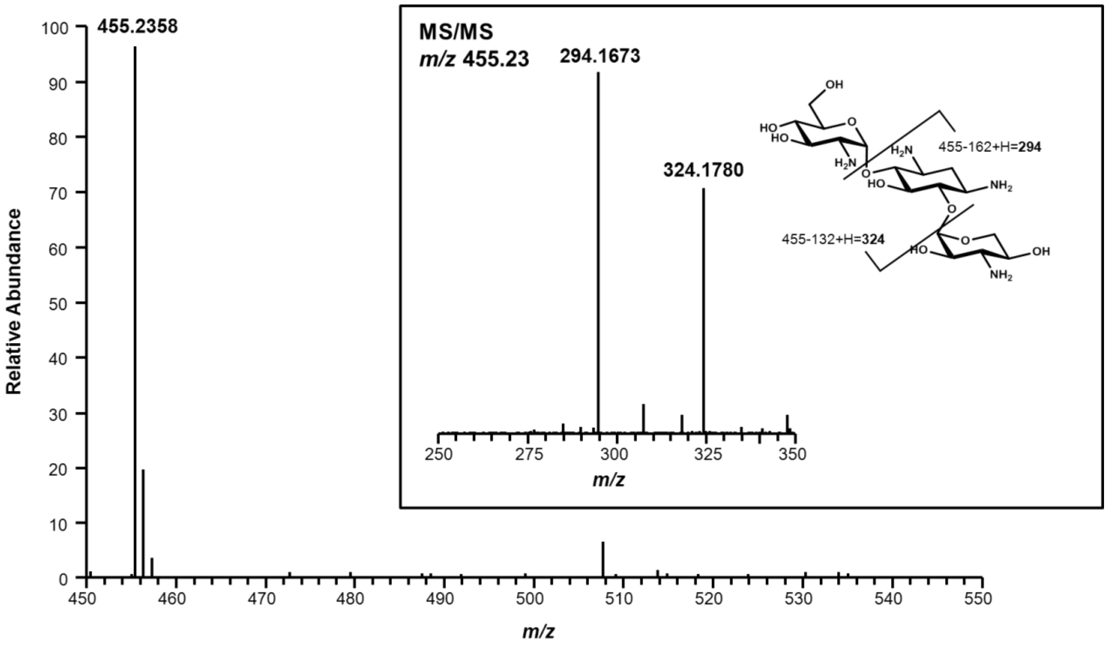

455.2358
Relative Abundance
m/z
MS/MS
m/z 455.23
294.1673
324.1780
455-162+H=294
455-132+H=324

D

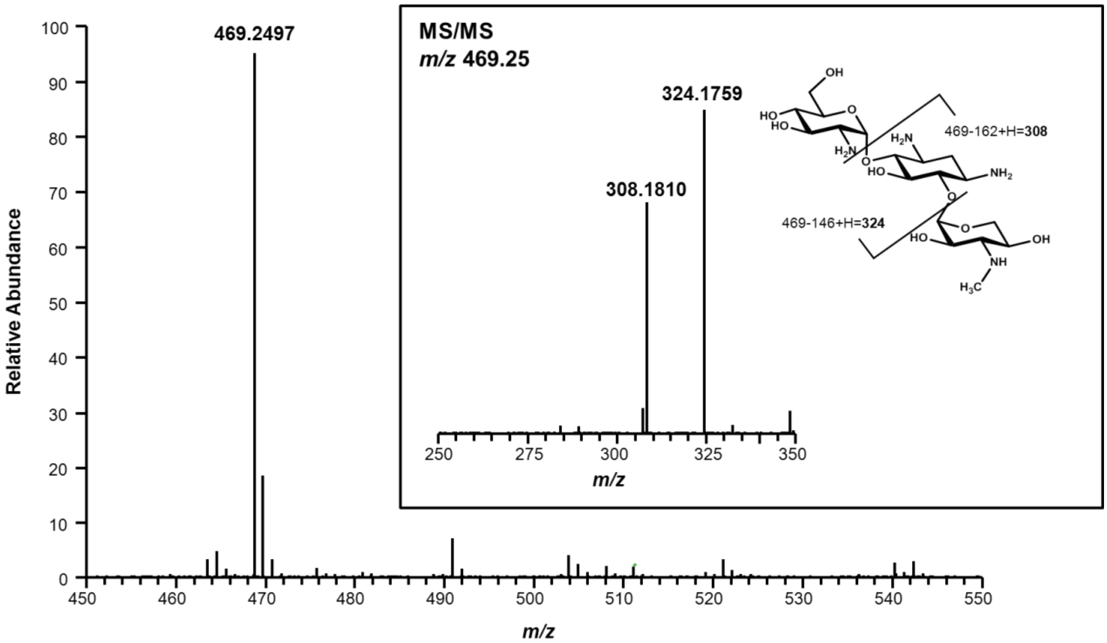

469.2497
Relative Abundance
m/z
MS/MS
m/z 469.25
324.1759
308.1810
469-162+H=308
469-146+H=324

E

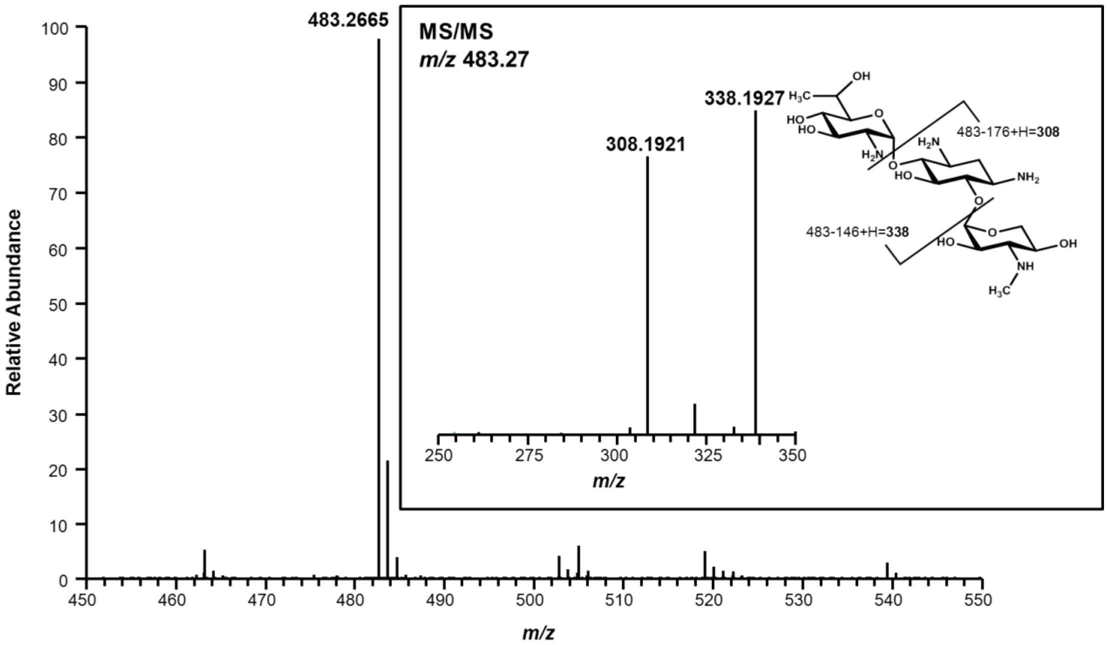

483.2665
Relative Abundance
m/z
MS/MS
m/z 483.27
338.1927
308.1921
483-176+H=308
483-146+H=338

Figure S3. LC-ESI-HRMS analysis of the production of the gentamicin C complex and gentamicin-related metabolites by *Micromonospora echinospora* mutants when fed with gentamicin A or G418. Related to Figure 2.

Total ion current trace of (A) gentamicin standard; and fermentation culture extracts from (B) wild-type; (C) ΔgenD2 mutant fed with gentamicin A; (D) ΔgenS2 mutant fed with gentamicin A; (E) ΔgenN mutant fed with gentamicin A; (F) ΔgenD1 mutant fed with gentamicin A; (G) ΔgenD1 mutant fed with G418 (**5**).

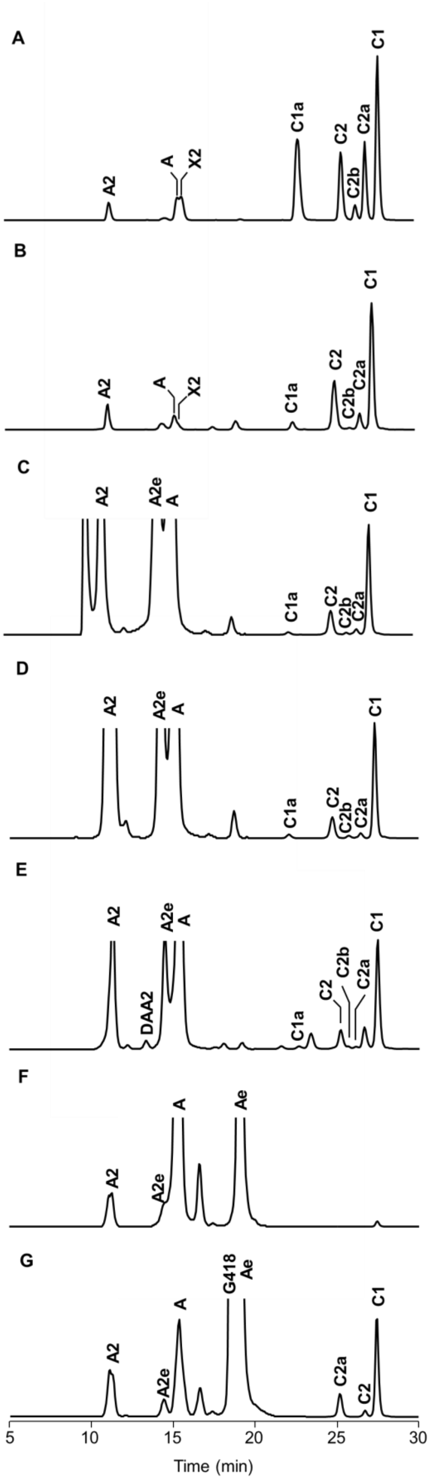

Figure S4. Characterization of purified recombinant GenD2, GenS2, GenN and GenD1. Related to Figure 3 and 4.

(A) SDS-PAGE gel of GenD2, GenS2, GenN and GenD1; (B) UV-visible absorption spectra of GenD2, GenS2 and GenD1.

A

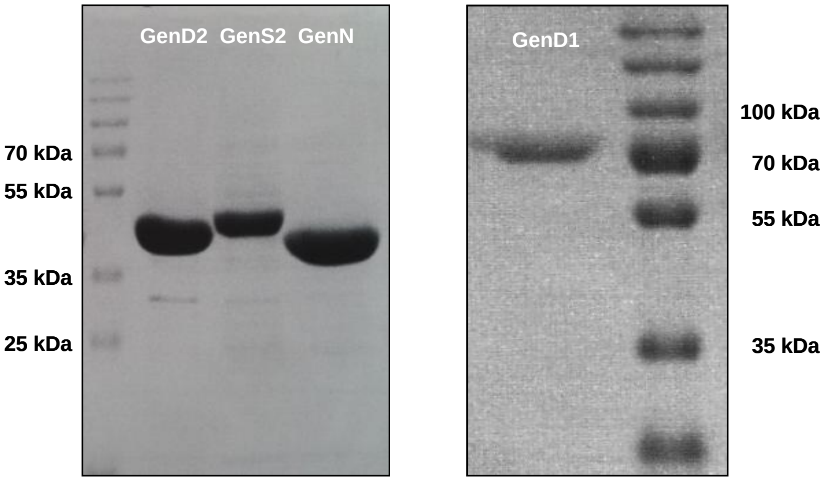

B

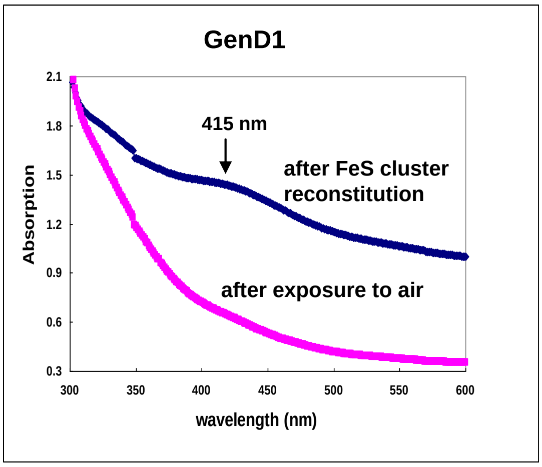

Figure S5. LC-ESI-HRMS analysis of GenD2-, GenS2- and GenN-catalyzed modifications on kanamycin B and tobramycin. Related to Figure 3.

Selective ion monitoring was carried out on

(A) $[M+H]^+$ (m/z 484) and $[M+Na]^+$ (m/z 506) ions of kanamycin B (**11**);

(B) $[M+H]^+$ (m/z 483) and $[M+Na]^+$ (m/z 505) ions of 3''-deamino-3''-oxo-kanamycin B (**13**), the product of GenS2 catalyzed de-amination of **11**;

(C) $[M+H]^+$ (m/z 485) and $[M+Na]^+$ (m/z 507) ions of 3''-deamino-3''-hydroxy-kanamycin B (**15**), the product of GenD2 catalyzed hydrogenation of **13**;

(D) $[M+H]^+$ (m/z 498) and $[M+Na]^+$ (m/z 520) ions of 3''N-methyl-kanamycin B (**17**), the product of GenN catalyzed methylation of **11**.

(E) $[M+H]^+$ (m/z 468) and $[M+Na]^+$ (m/z 490) ions of tobramycin (**12**);
(F) $[M+H]^+$ (m/z 467) and $[M+Na]^+$ (m/z 489) ions of 3''-deamino-3''-oxo-tobramycin (**14**), the product of GenS2 catalyzed de-amination of **12**;
(G) $[M+H]^+$ (m/z 469) and $[M+Na]^+$ (m/z 491) ions of 3''-deamino-3''-hydroxy-tobramycin (**16**), the product of GenD2 catalyzed hydrogenation of **14**;
(H) $[M+H]^+$ (m/z 482) and $[M+Na]^+$ (m/z 504) ions of 3''N-methyl-tobramycin (**18**), the product of GenN catalyzed methylation of **12**.
MS/MS analysis of $[M+H]^+$ (m/z 484), $[M+H]^+$ (m/z 483), $[M+H]^+$ (m/z 485), $[M+H]^+$ (m/z 498) MS^2 analysis of $[M+H]^+$ (m/z 468), $[M+H]^+$, (m/z 467), $[M+H]^+$, (m/z 485) and $[M+H]^+$ (m/z 482) ions are also shown.

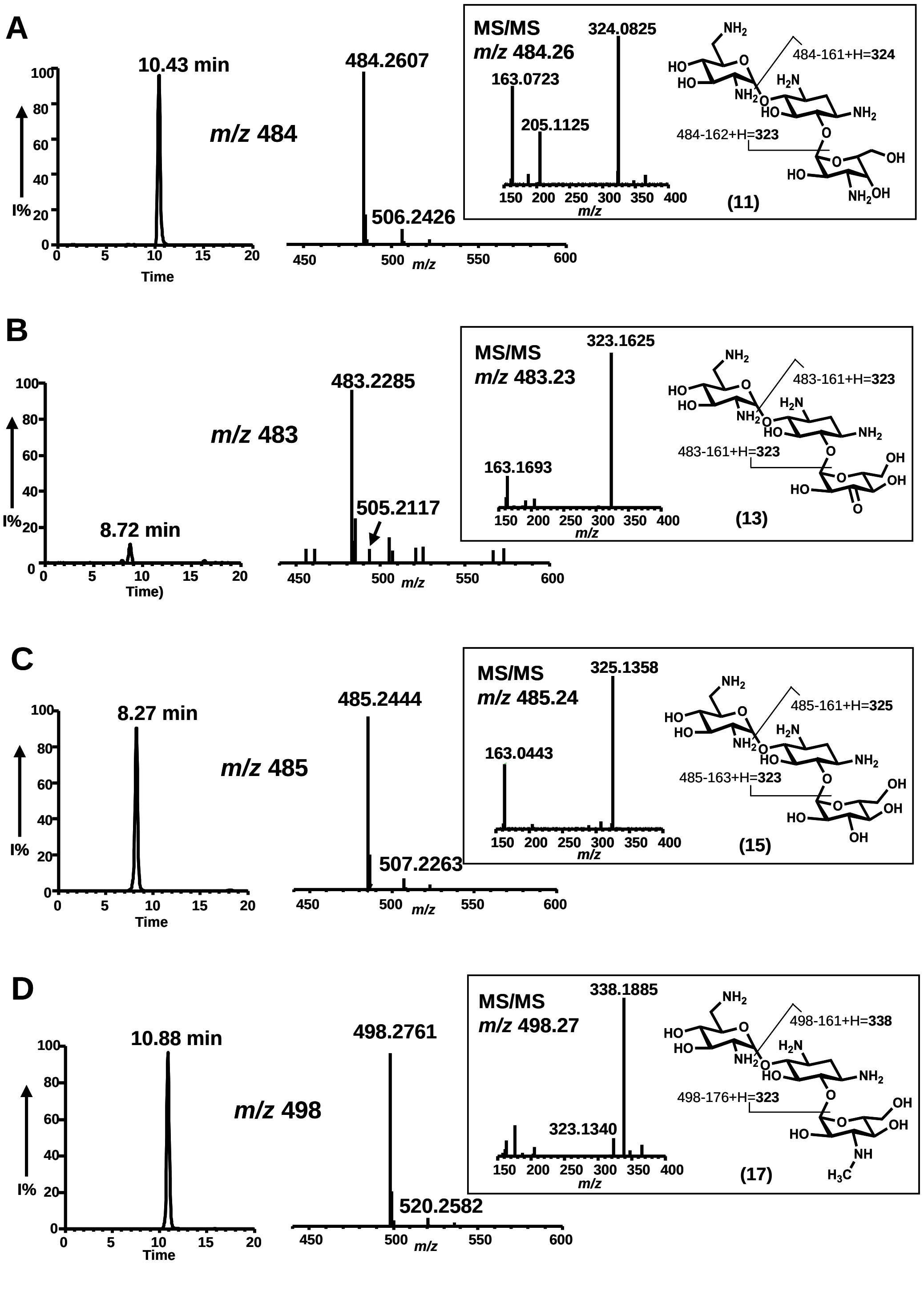

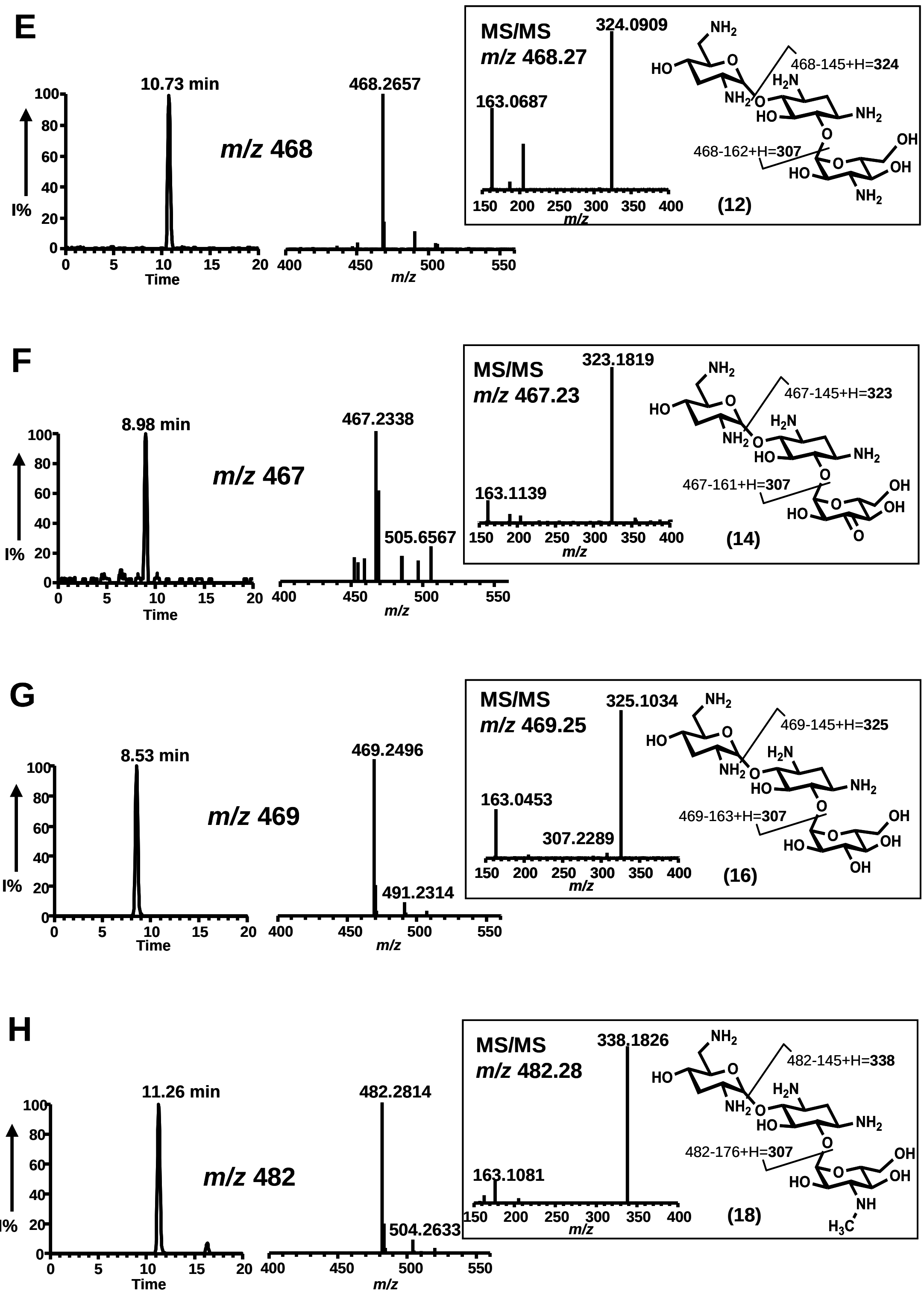

E
10.73 min
468.2657
m/z 468
I%
Time
m/z
MS/MS
m/z 468.27
324.0909
163.0687
468-145+H=324
468-162+H=307
(12)
F
8.98 min
467.2338
505.6567
m/z 467
I%
Time
m/z
MS/MS
m/z 467.23
323.1819
163.1139
467-145+H=323
467-161+H=307
(14)
G
8.53 min
469.2496
491.2314
m/z 469
I%
Time
m/z
MS/MS
m/z 469.25
325.1034
163.0453
307.2289
469-145+H=325
469-163+H=307
(16)
H
11.26 min
482.2814
504.2633
m/z 482
I%
Time
m/z
MS/MS
m/z 482.28
338.1826
163.1081
482-145+H=338
482-176+H=307
(18)

Supplemental Experimental Procedures

1. List of oligonucleotide primers used in this work

Primer	Oligonucleotide sequences (5' to 3')	Restriction Site
genD2-L1	CGC**CATATG**AGAGATGGAACTGGC	*Nde*I
genD2-L2	CCG**GAATTC**CACTCGGGGATC	*Eco*RI
genD2-R1	GTG**GAATTC**AATGCCTGACAACAAG	*Eco*RI
genD2-R2	CAG**AAGCTT**CTCGCCCTCCCG	*Hin*dIII
genD2-CK1	GCTGCGGTTCGACAACAAGC	
genD2-CK2	TTGGACGGGATCGGCAGCAC	
genS2-L1	CGT**CATATG**TGTCGCATTCCCACCG	*Nde*I
genS2-L2	GGC**GAATTC**CTGGTGCATGGTGTTC	*Eco*RI
genS2-R1	GAG**GAATTC**CAGGACATGCTGGATG	*Eco*RI
genS2-R2	CTG**AAGCTT**TACAACATCGGCCAGG	*Hin*dIII
genS2-CK1	TGGAGAACTACTGGGTGAAGCA	
genS2-CK2	TCGACCGTGACCTTGAGGAA	
genD2-a	CACGG**CATATG**CTGCCGATGG	*Nde*I
genD2-b	GGGG**GAATTC**TTGTCAGGCATTCAT	*Eco*RI
genS2-a	CCG**CATATG**ACGCAGAAACTGGCCA	*Nde*I
genS2-b	GCCA**GAATTC**GATCATAGGCTCTTC	*Eco*RI
genK-CK1	CGGGCGAACCTTCGGGATA	
genK-CK2	CCGTCAGCGTTGGCAATAA	
genD1-L1	GGC**CATATG**GCTCGCGGCCG	*Nde*I
genD1-L2	AAG**GAATTC**CGTGAGGGTCGCCACC	*Eco*RI
genD1-R1	CCG**GAATTC**GCCCTCGGGGC	*Eco*RI
genD1-R2	GTG**AAGCTT**GATCGGCCGGACATCG	*Hin*dIII
genD1-CK1	GAAGCTCGCCGATGCCA	
genD1-CK2	CAGGTGAAGGCGGTGGTG	
genD1-a	CGC**CATATG**ACCGTCACTAACAAG	*Nde*I
genD1-b	CCG**GAATTC**TCAGCGGCTACCTGCCCC	*Eco*RI

genN-L1	GCG**<u>CATATG</u>**CTCGTAGACCCAGTTC	*Nde*I
genN-L2	CTG**GAATTC**CGAGCCTCCGACGATC	*Eco*RI
genN-R1	GAC**GAATTC**CTGCGGGGCTGACCCC	*Eco*RI
genN-R2	GAG**AAGCTT**GCCGCCGACTCCGACC	*Hin*dIII
genN-CK1	GGATGGGATGCCAACGACC	
genN-CK2	ACCGCGACGACGATGACG	
genN-a	CGC**CATATG**ATCGTCGGAGGCTCG	*Nde*I
genN-b	CCG**GAATTC**TCAGCCCCGCATGAGCCG	*Eco*RI
pGenD1-For	GGAGTCCT**CATATG**ACCGTCACTAACAAGA	*Nde*I
pGenD1-Rev	5GGCCAGGGCC**GGATCC**GGACGGGGTCGCCA	*Bam*HI
pGenD2-For	GTGGGTGCTG**CATATG**GTTG AGCGCCTGGG	*Nde*I
pGenD2-Rev	CCCAACACGT**GAATTC**CGCCCATCGGGTCG	*Eco*RI
pGenN-For	ACTCTCGGGAGTAG**CATATG**ATCGTCGGAG	*Nde*I
pGenN-Rev	GGTGCGGT**GGATCC**AACCTG TGGCAGGGCC	*Bam*HI
pGenS2-For	GGCAG GTAGCCG**CATATG**ACGCAGAAACTG	*Nde*I
pGenS2-Rev	TCGCCGATCGG**GAATTC**TTCGAGGGATCGG	*Eco*RI

Primer pairs for amplification of left- or right-flanking fragments of a target gene, for PCR/sequencing confirmation, for complementation plasmid construction, and for cloning target genes for over-expression are marked with suffixes –L1/–L2, -R1/-R2, –a/-b, CK1/CK2, or –For/-Rev, respectively.

2. List of bacterial strains and plasmids used in this study

Strain/Plasmid	Characteristics	Reference
E.coil		
DH10B	Host for general cloning	Invitrogen
NovaBlue	Host for general cloning	Novagen
BL21(DE3)	Host for recombinant protein expression	Novagen
ET12567/pUZ8002	Donor strain for conjugation between *E.coli* and *Streptomyces*	MacNeil et al., (1992)
Micromonospora echinospora		
ATCC15835	Gentamicin producing wild-type strain	Weinstein et al., (1963)

ΔgenD2	*genD2* single in-frame deletion mutant	This work
ΔgenS2	*genS2* single in-frame deletion mutant	This work
ΔgenD2ΔgenK	*genD2* and *genK* in-frame deletion mutant	This work
ΔgenS2ΔgenK	*genS2* and *genK* in-frame deletion mutant	This work
ΔgenD2::*genD2*	Self-complementation of *genD2* in ΔgenD2	This work
ΔgenD1::*genD1*	Self-complementation of *genD1* in ΔgenD1	This work
ΔgenS2::*genS2*	Self-complementation of *genS2* in ΔgenS2	This work
ΔgenN::*genN*	Self-complementation of *genN* in ΔgenN	This work
ΔgenD2ΔgenK::*genK*	Self-complementation of *genK* in ΔgenD2ΔgenK	This work
ΔgenS2ΔgenK::*genK*	Self-complementation of *genK* in ΔgenS2ΔgenK	This work
**Plasmid**		
pUC18	Vector for sub-cloning and DNA sequencing	Takara
pYH7	*E. coli-Streptomyces* shuttle vector	Sun et al., (2009)
pWHU1	*genK* in-frame deletion construct	Guo et al. (2014)
pWHU6	*genD2* in-frame deletion construct	This work
pWHU21	*genS2* in-frame deletion construct	This work
pYH287	*genD1* in-frame deletion construct	This work
pYH289	*genN* in-frame deletion construct	This work
pWHU67	*genK* self-complementation construct	Guo et al., (2014)
pWHU115	*genS2* self-complementation construct	This work
pWHU184	*genD2* self-complementation construct	This work
pWHU66	*genD1* self-complementation construct	This work
pWHU68	*genN* self-complementation construct	This work
pET28/genD2	for over-expression of recombinant GenD2	This study
pET28/genS2	for over-expression of recombinant GenS2	This study
pET28/genN	for over-expression of recombinant GenN	This study
pET28/genD1	for over-expression of recombinant GenD1	This study
pDB1282	for over-expression of iron-sulfur cluster (isc) biosynthetic genes	Zheng et al., 1998

3. Structures of 3''-dehydro-3''-amino-gentamicin A2 and its homologs, kanamycin B, tobramycin and sisomicin.

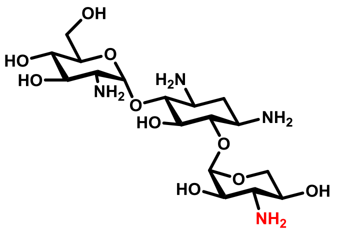

3''-dehydro-3''-amino-gentamicin A2
(DAA2, 9)

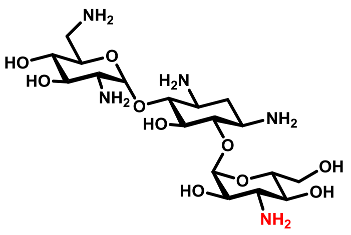

kanamycin B (11)

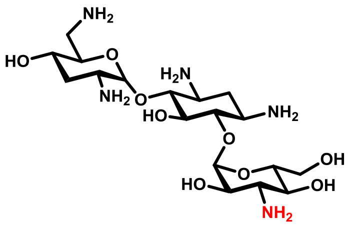

tobramycin (12)

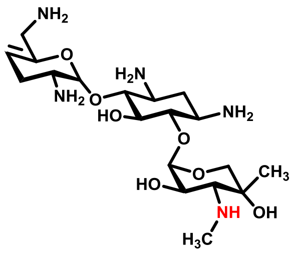

sisomicin (19)

4. Purification of gentamicin A2

Purification of gentamicin A2 (**3**) from extracts of the ΔgenS2ΔgenK mutant fermentation broth after cation exchange (see Experimental Procedures) was performed on a Waters 2535 semi-preparative HPLC system using a ZORBAX SB-C18 (9.4 × 250 mm, 5μ, Agilent) semi-preparative column, with as mobile phase (A) 0.2% TFA in water adjusted to pH 2 with NH_4OH and (B) acetonitrile. Elution of gentamicin A2 was carried out isocratically at 95% A for 15 min at a flow rate of 4 ml/min. The column eluate was directed to an Evaporative Light Scattering Detector(ELSD, Alltech 3300) with 2ml/min splitting, and the rest of the eluate was collected in 1 ml fractions. The atomization temperature of ELSD was 55°C and the drift tube temperature was 48 °C. Fractions containing gentamicin A2 were pooled and further confirmed by LC-ESI-HRMS.

5. Kinetic study of GenN with kanamycin B

8.5 nM GenN was incubated in 500 μl Tris-HCl (50 mM, pH 7.5) buffer at 30°C with kanamycin B (**11**) at concentrations of 30, 40, 50, 60 and 70 μM in the presence of 250 μM SAM. An 80 μl aliquot of each reaction was taken out after 1, 2, 3, 4, 5 and 6 min incubation and mixed immediately with an equal volume of ice cold chloroform to precipitate the enzyme. The reactions were analyzed by LC-ESI-MS. The product 3''N-methylkanamycin B (**17**) was quantified

based on the peak integration of the selected ion. Data points are the mean value of three replicates.

6. Conditions for LC-ESI-MS analyses

LC-ESI-HRMS analysis of gentamicin-related metabolites and in vitro assays were performed on a Thermo LTQ-Orbitrap XL instrument using a Luna C18 column (250 mm × 4.6 mm, 5 μ, Phenomenex) with a flow rate of 0.4 ml/min. LC-MS analysis of proteins was carried out on a ThermoFinnigan LCQ fitted with an ESI source connected to an Agilent HP 1100 HPLC system using a Nucleosil C4 column (250 mm × 2 mm, 5 μ, Macherey-Nagel). The mobile phase and gradients used for HPLC are as follows:

HPLC gradient used for analysis of gentamicin-related metabolites

Mobile phase A: 0.2% TFA in water; Mobile phase B: acetonitrile. 2% B to 14% B over 18 min, then to 90 % B within 1 min, maintained at 90% B for 5 min, returned to 2% B over 1 min and maintained at 2% B for a further 5 min. The injection volume was 5 μl for each gentamicin standard (500 μg ml^{-1}) and 20 μl for each sample. Each cultivation and analysis was performed in triplicate.

HPLC gradient for analysis of aminoglycosides in enzymatic assays

Mobile phase A: 0.2% TFA in water; Mobile phase B: 0.1% TFA in acetonitrile. 2%B to 8%B over 9 min then to 90%B over 1 min, maintained at 90%B for 4 min, returned to 2%B over 1 min and then maintained at 2%B for a further 5 min. flow rate 0.6 ml/min.

HPLC gradient for analysis of 5’-deoxydenosine in enzymatic assays

Mobile phase A: 0.1% TFA in water; Mobile phase B: 0.1% TFA in acetonitrile. 0%B to 20% B over 30 min. 0.6 ml/min.

HPLC gradient for analysis of proteins

Mobile phase A: 0.1% TFA in water; Mobile phase B: 0.1% TFA in acetonitrile. 35% B to 45 % B over 5 min, increasing gradient of B to 75% over 20 min, gradient to 95% within 2 min, maintained at 95% B for 7 min, followed by a gradient from 95% B to 35%B within 3 min;

References

1. Kim J.Y., Suh J.W., Kang S.H., Phan T.H., Park S.H., Kwon H.J.(2008). Gene inactivation study of gntE reveals its role in the first step of pseudotrisaccharide modifications in gentamicin biosynthesis. Biochem. Biophys. Res. Commun. *372*, 730-734.

2. MacNeil, D.J., Occi, J.L., Gewain, K.M., MacNeil, T., Gibbons, P.H., Ruby, C.L., and Danis, S.J. (1992). Complex organization of the *Streptomyces avermitilis* genes encoding the avermectin polyketide synthase. Gene *115*,

119-125.

3. Weinstein, M.J., Luedemann, G.M., Oden, E.M., Wagman, G.H., Rosselet, J.P., Marquez, J.A., Coniglio, C.T., Charney, W., Herzog, H.L., and Black, J (1963). Gentamicin, a new antibiotic complex from *Micromonospora*. J. Med. Chem. *6*, 463-464.

4. Sun, Y., He, X., Liang, J., Zhou X., and Deng, Z. (2009). Analysis of functions in plasmid pHZ1358 influencing its genetic and structural stability in *Streptomyces lividans* 1326. Appl. Microbiol. Biotechnol. *82*, 303-310.

5. Guo, J., Huang, F., Huang, C., Duan, X., Jian, X., Leeper, F., Deng, Z., and Leadlay, P.F., Sun, Y. (2014). Specificity and promiscuity at the branch point in gentamicin biosynthesis. Chem Biol. *21*, 608-618.
