# Supplementary material for: Delineating the Biosynthesis of Gentamicin X2, the Common Precursor of the Gentamicin C Antibiotic Complex
Source: Chem Biol. 2015 Feb 19;22(2):251–61. doi: 10.1016/j.chembiol.2014.12.012 (PMC4340712; doi:10.1016/j.chembiol.2014.12.012)
Supplement: Document S2. Article plus Supplemental Information [file mmc2.pdf]

Article

Chemistry & Biology

Delineating the Biosynthesis of Gentamicin X2, the Common Precursor of the Gentamicin C Antibiotic Complex

Highlights

- Key portion of the pathway to a globally used antibiotic is defined
- Four enzymes convert gentamicin A2 into gentamicin X2
- GenD1 is an unusual cobalamin- and radical SAM-dependent methyltransferase

Authors

Chuan Huang, Fanglu Huang, ...,
Peter F. Leadlay, Yuhui Sun

Correspondence

pfl10@cam.ac.uk (P.F.L.),
yhsun@whu.edu.cn (Y.S.)

In Brief

All five components of the clinically valuable antibiotic gentamicin C complex derive from the pseudotrisaccharide gentamicin X2. Huang et al. use gene knockouts and in vitro reconstitution to identify the four pathway enzymes catalyzing the final steps leading to this key intermediate.

Huang et al., 2015, Chemistry & Biology *22*, 251–261
February 19, 2015 ©2015 The Authors
http://dx.doi.org/10.1016/j.chembiol.2014.12.012

CellPress

Delineating the Biosynthesis of Gentamicin X2, the Common Precursor of the Gentamicin C Antibiotic Complex

Chuan Huang,[1,4] Fanglu Huang,[2,4] Eileen Moison,[2] Junhong Guo,[1] Xinyun Jian,[1] Xiaobo Duan,[1] Zixin Deng,[1,3] Peter F. Leadlay,[2,*] and Yuhui Sun[1,*]
[1]Key Laboratory of Combinatorial Biosynthesis and Drug Discovery, Ministry of Education, and Wuhan University School of Pharmaceutical Sciences, Wuhan University, Wuhan 430071, People's Republic of China
[2]Department of Biochemistry, University of Cambridge, Cambridge CB2 1GA, UK
[3]Hubei Engineering Laboratory for Synthetic Microbiology, Wuhan Institute of Biotechnology, Wuhan 430075, People's Republic of China
[4]Co-first author
*Correspondence: pfl10@cam.ac.uk (P.F.L.), yhsun@whu.edu.cn (Y.S.)
http://dx.doi.org/10.1016/j.chembiol.2014.12.012
This is an open access article under the CC BY license (http://creativecommons.org/licenses/by/4.0/).

SUMMARY

Gentamicin C complex is a mixture of aminoglycoside antibiotics used worldwide to treat severe Gram-negative bacterial infections. Despite its clinical importance, the enzymology of its biosynthetic pathway has remained obscure. We report here insights into the four enzyme-catalyzed steps that lead from the first-formed pseudotrisaccharide gentamicin A2 to gentamicin X2, the last common intermediate for all components of the C complex. We have used both targeted mutations of individual genes and reconstitution of portions of the pathway in vitro to show that the secondary alcohol function at C-3″ of A2 is first converted to an amine, catalyzed by the tandem operation of oxidoreductase GenD2 and transaminase GenS2. The amine is then specifically methylated by the *S*-adenosyl-L-methionine (SAM)-dependent *N*-methyltransferase GenN to form gentamicin A. Finally, C-methylation at C-4″ to form gentamicin X2 is catalyzed by the radical SAM-dependent and cobalamin-dependent enzyme GenD1.

INTRODUCTION

Gentamicins are clinically valuable aminoglycoside antibiotics isolated as gentamicin C complex, a mixture of five components (Figure 1), from the filamentous bacterium *Micromonospora echinospora*. Gentamicins are protein synthesis inhibitors used to combat Gram-negative bacterial infections. They are also being explored in other therapeutic areas (Hainrichson et al., 2008; Linde and Kerem, 2008; Cuccarese et al., 2013). However, gentamicins carry a serious risk of kidney damage and hearing loss (Bockenhauer et al., 2009) which limits their utility, and it is therefore encouraging that evidence is available that an individual component of the gentamicin mixture may have lower toxicity (Sandoval et al., 2006; Kobayashi et al., 2008). This makes the gentamicin biosynthetic pathway an attractive target for reengineering to favor a specific component.

Gentamicins are modified sugars, characteristically containing an unusual aminocyclitol ring (2-deoxystreptamine, 2-DOS [**1**]) (Houghton et al., 2010; Park et al., 2013). The outlines of the gentamicin pathway have been known for some time (Testa and Tilley, 1975, 1976; Kase et al., 1982a, 1982b), and in the wake of the comprehensive sequencing of aminoglycoside biosynthetic gene clusters (Ota et al., 2000; Unwin et al., 2004; Kharel et al., 2004a, 2004b, 2004c; Huang et al., 2005; Subba et al., 2005; Aboshanab, 2005; Hong et al., 2009) rapid progress has been made in identifying the enzymatic steps that lead to the 2-DOS scaffold, and thence to the pseudodisaccharide paromamine (**2**) (Llewellyn and Spencer, 2006; Thibodeaux et al., 2008; Kudo and Eguchi, 2009; Kudo et al., 2009) and the pseudotrisaccharide gentamicin A2 (**3**) (Park et al., 2008) (Figure 1). This has enabled a sharper focus on those genes likely to govern the later steps of gentamicin biosynthesis.

We (Guo et al., 2014) and others (Karki et al., 2012; Hong and Yan, 2012; Li et al., 2013) have used specific gene deletions to probe the identity of the enzymes that act on gentamicin X2 (**4**), partitioning this intermediate between a methylated branch that gives rise via G418 (**5**) to gentamicin components C2a, C2, and C1 and an unmethylated branch that gives rise to C1a and C2b (Figure 1). The cluster contains multiple, mutually homologous genes for methyltransferases, oxidoreductases, and aminotransferases with potential for overlapping substrate specificities, but by combining analysis of specific gene deletions with in vitro reconstitution of the enzymes involved in dehydrogenation/transamination of G418 (**5**) (Guo et al., 2014) it has been possible to deconvolute the specific roles of some of the individual genes in this final stage of the pathway. Also, Liu and colleagues have demonstrated the activity in vitro of GenK, which methylates **4** to produce **5**, and have confirmed that it is a cobalamin-dependent radical *S*-adenosyl-L-methionine (SAM) enzyme (Kim et al., 2013).

Here we present a similar in vivo and in vitro analysis to define the enzymology of the pathway from the first pseudotrisaccharide **3** to the last common precursor of the gentamicin C complex, gentamicin X2 (**4**). It has already been established that disruption

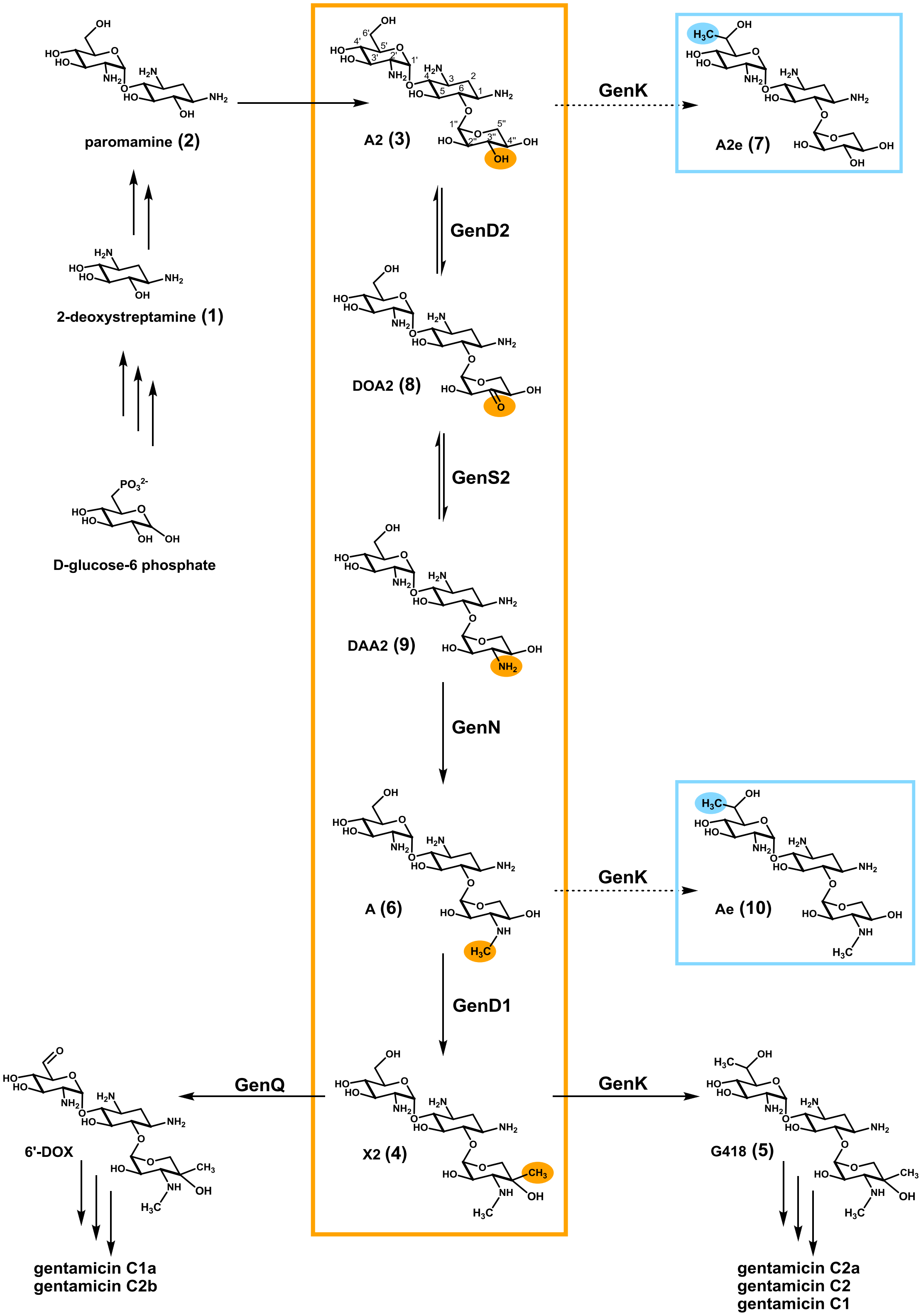

Figure 1. The Pathway to Gentamicin X2

The roles revealed in this study of GenD2, GenS2, GenN, and GenD1 are highlighted in orange. Shunt products of GenK are shown in blue. For details, see the text. A2 (**3**): gentamicin A2; DOA2 (**8**): 3″-dehydro-3″-oxo-gentamicin A2; DAA2 (**9**): 3″-dehydro-3″-amino-gentamicin A2; X2 (**4**): gentamicin X2; A2e (**7**): 6′-methylgentamicin A2; Ae (**6**): 6′-methylgentamicin A. Carbon numbering is shown with the structure of **3**.

of the gene *genD1*, also known as *gntE* (Unwin et al., 2004) or *gtmI* (Kharel et al., 2004c), by a thiostrepton resistance gene (*tsr*) leads to accumulation of **3** (Figure 1) (Kim et al., 2008). These authors interpreted their result as meaning that GenD1 catalyzes *N*-methylation of the 3″-amino group in **3**; but several alternative explanations of this result are possible, especially as polar effects on the downstream *genS2* gene were not ruled out. By analysis of the gentamicin-related metabolites accumulated in strains bearing single and multiple specific gene deletions, by following the bioconversion of specific intermediates fed to such mutants, and by reconstituting all of the steps using purified recombinant enzymes, we demonstrate here that in fact the respective (and essential) catalytic contributions of the dehydrogenase GenD2, the pyridoxal phosphate-dependent aminotransferase GenS2, the SAM-dependent *N*-methyltransferase GenN, and the radical SAM-dependent C-methyltransferase GenD1 are as shown in Figure 1. We also provide evidence for the ability of GenK to act precociously on **3** and gentamicin A (**6**) (Figure 1) to generate shunt metabolites.

RESULTS

GenD2, GenS2, and GenN Are Essential for Conversion of Gentamicin A2 into Gentamicin A

The gene *genD2* (also known as *gntC* [Unwin et al., 2004] or *gtmC* [Kharel et al., 2004c]) is a candidate to act on **3** because it is predicted to encode an NAD(P)H-linked dehydrogenase with significant sequence identity to Sis12, KanD2, and TobD2, enzymes catalyzing similar reactions in other aminoglycoside pathways. To investigate the proposed role of the GenD2 dehydrogenase in the specific oxidation of **3** at the C-3″-hydroxyl, this gene was knocked out by targeted in-frame deletion of a 930 bp internal fragment (Figure S1A available online). The mutant was confirmed by PCR and Southern hybridization (Figure S1A). Liquid chromatography/electrospray ionization/high-resolution mass spectrometry (LC-ESI-HRMS) analysis confirmed that fermentation of this mutant produces elevated levels of **3**, but no other gentamicins normally seen in the wild-type (Figures 2C and S2A). A second new species was also observed, which from LC-ESI-HRMS and tandem mass spectrometry (MS/MS) analysis (Figure S2B) appears to represent a derivative of **3** methylated at the C-6′ position. This species was first detected by Kim et al. (2008) as a by-product of their *genD1* disruptant and was named by them gentamicin A2e (**7**; Figure 1) but not characterized. Complementation of the ΔgenD2 mutant, carried out by using plasmid pWHU184 containing *genD2* under the control of the P*ermE** promoter (Figure S1G), restored the production of gentamicin C complex and of various intermediates to wild-type levels (Figure 2G). Chemical complementation of the ΔgenD2 mutant by feeding **6** also similarly restored production of gentamicin C complex (Figure S3C), confirming the primary role of GenD2 in the section of the pathway between **3** and **6**.

Recent in vivo (Kim et al., 2008; Hong and Yan, 2012; Guo et al., 2014) and in vitro (Kim et al., 2013) work has unequivocally established that methylation at the C-6′ position is normally carried out on **4** by the SAM-dependent methyltransferase GenK. We suspected therefore that the appearance of **7** in our ΔgenD2 mutant was a consequence of the action of GenK in the absence of its normal substrate. To test this, a double mutant deleted in-frame in both *genD2* and *genK* was constructed (Figure S1C). LC-ESI-HRMS analysis confirmed that this mutant accumulated **3** but not **7** (Figure 2E). Complementation of the ΔgenD2ΔgenK strain with plasmid pWHU67 containing *genK* under the control of the P*ermE** promoter (Figure S1K), restored the coproduction of the shunt product **7** (Figure 2I).

GenS2, the product of the unassigned gene *genS2* (also known as *gntF* [Unwin et al., 2004] or *gtmD* [Kharel et al., 2004c]) is a plausible candidate to partner GenD2 in amination at C-3″ because it is predicted to encode a pyridoxal phosphate-dependent aminotransferase with significant sequence identity to Sis15, KanS2, and TobS2, enzymes catalyzing similar reactions in other aminoglycoside pathways. Accordingly, this gene was knocked out by targeted in-frame deletion and the mutant was confirmed by PCR and Southern hybridization (Figure S1B). LC-ESI-HRMS analysis confirmed that fermentation of this mutant only produced **3** and **7** (Figure 2D). Complementation of the ΔgenS2 mutant, carried out by using plasmid pWHU115 containing *genS2* under the control of the P*ermE** promoter (Figure S1H), restored the production of gentamicin C complex and of various intermediates to wild-type levels (Figure 2H). Chemical complementation of the ΔgenS2 mutant by feeding **6** also similarly restored production of gentamicin C complex (Figure S3D), confirming that the *genS2* gene, like *genD2*, is essential and acts exclusively in the section of the pathway between **3** and **6**, consistent with a specific role in partnering GenD2 to accomplish amination at C-3″. Also, the shunt product gentamicin **7** was not present in a ΔgenS2ΔgenK double mutant (Figures 2F and S1D) but its appearance was restored by complementation in *trans* by constitutive expression of *genK* (Figures 2J and S1L). The presumed intermediate in amination at C-3″ is 3″-dehydro-3″-oxo-gentamicin A2 (DOA2 [**8**]; Figure 1), and this was not detected in the ΔgenS2 or ΔgenS2ΔgenK mutants. Either the compound is unstable, or the GenD2-catalyzed oxidation does not proceed if the ketone product is not continuously removed (see below).

The third and final step between **3** and the known intermediate **6** involves *N*-methylation of the newly introduced 3″-amino group in the garosamine ring (Figure 1). Of the several predicted methyltransferase genes in the gentamicin gene cluster, *genN* (GI: 85814038; Aboshanab, 2005) is an attractive candidate for this role. It is predicted to encode a conventional SAM-dependent methyltransferase, and its closest known homolog (90% sequence identity) is Sis30 in the cluster for sisomicin ([**19**]; Hong et al., 2009), a closely related aminoglycoside which requires a similar *N*-methylation (Supplemental Experimental Procedures). The gene clusters for the aminoglycosides kanamycin (**11**) and tobramycin (**12**), which do not contain a 3″-*N*-methyl group (Supplemental Experimental Procedures), contain no gene homologous to GenN.

GenN was knocked out by targeted in-frame deletion of a 933 bp internal fragment (Figure S1E). LC-ESI-HRMS analysis revealed that fermentation of this mutant produced **3** and **7** (Figure 2L), together with small amounts of a compound with the predicted mass of 3″-dehydro-3″-amino-gentamicin A2 (DAA2 [**9**]; Figures 1 and S2C), but none of the gentamicin C components was detected. Trace amounts of demethylgentamicin C1, demethylgentamicin C2, and demethylgentamicin C1a were observed. The fragmentation profiles of these demethylgentamicins

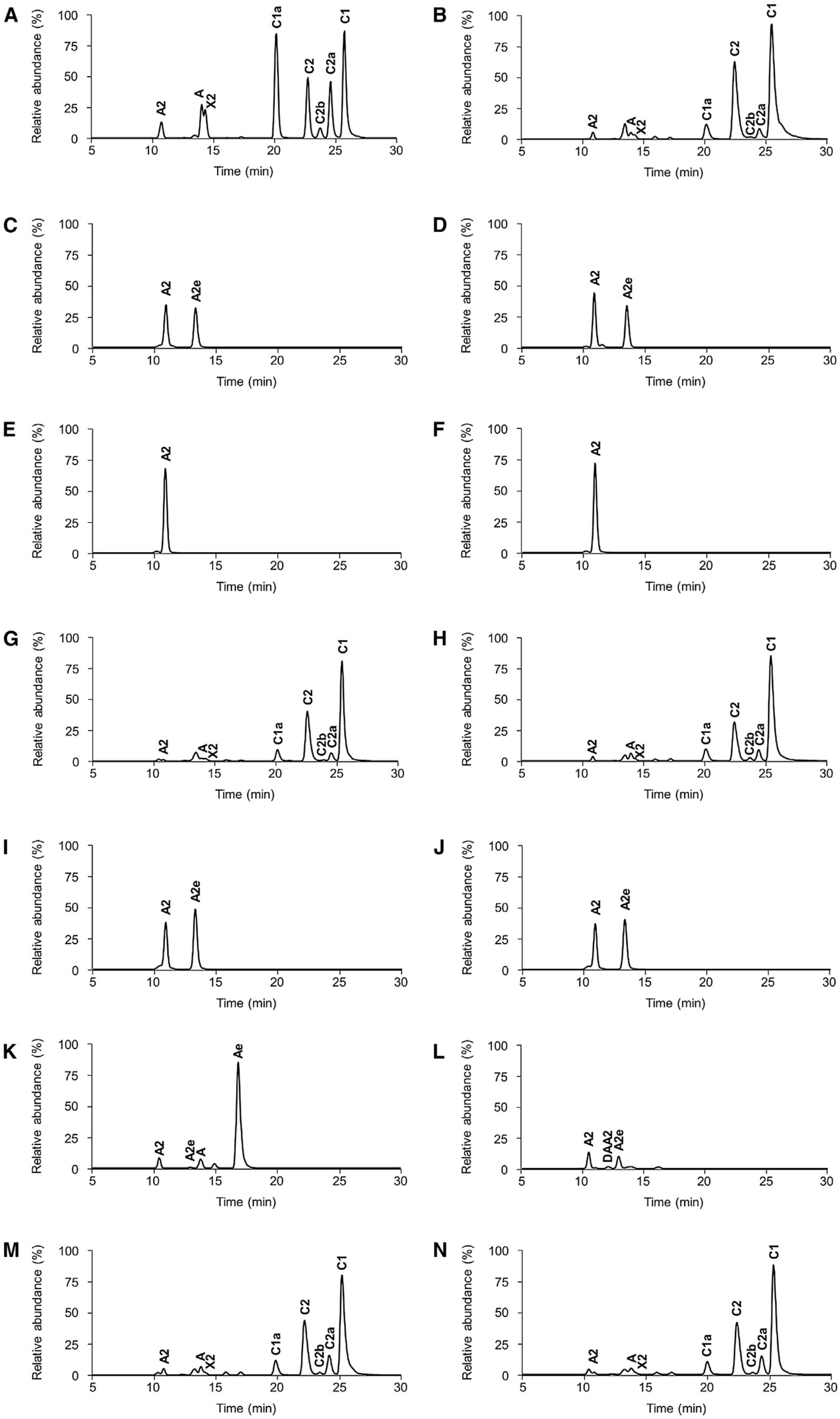

(legend on next page)

indicate that they lack a methyl group in the garosamine ring. Complementation of the ΔgenN mutant, carried out by using plasmid pWHU68 containing *genN* under the control of the P*ermE** promoter (Figure S1I), restored the production of gentamicin C complex and of various intermediates to wild-type levels (Figure 2N). Chemical complementation of the ΔgenN mutant by feeding gentamicin A restored production of gentamicin C complex (Figure S3E), confirming that the *genN* gene, like *genD2* and *genS2*, is essential and acts exclusively in the section of the pathway between **3** and **6**, consistent with a specific role in *N*-methylation of the C-3″ amino group.

The remaining step in the conversion of **3** to **4** is the C-methylation of **3** at the C-4″ position (Figure 1). The gene *genD1* (also known as *gntE* (Unwin et al., 2004) or *gtmI* (Kharel et al., 2004c), previously assigned as an oxidoreductase/methyltransferase (Kim et al., 2008), is a strong candidate to encode this methyltransferase, because it shows significant sequence identity to authentic cobalamin-dependent and radical SAM-dependent methyltransferases (Kim et al., 2013). Accordingly, the *genD1* gene was knocked out by in-frame deletion of an 1,851 bp internal fragment (Figure S1F). In contrast to previous findings based on disruption of the *genD1* gene (Kim et al., 2008), LC-ESI-HRMS analysis showed that fermentation of this mutant produced not only **3** and **7** but also **6** (Figures 2K and S2D). The most abundant peak was a new species with the mass of **4** but with a different retention time and MS/MS fragmentation pattern (Figure S2E). The appearance of this species (called here gentamicin Ae [**10**]) is consistent with GenK-catalyzed methylation of **6** at C-6′ in the absence of its normal gentamicin X2 (**4**) substrate. Complementation of the ΔgenD1 mutant in *trans*, using a plasmid housing *genD1* under a constitutive promoter (Figure S1J), restored wild-type levels of gentamicins (Figure 2M). Chemical complementation of the ΔgenD1 mutant by feeding **6** did not restore production of gentamicin C complex (Figure S3F), but complementation by feeding with **5** did restore the production of gentamicin C2a, C2, and C1, the products in the "methylated branch" (Figure S3G). This confirmed that the *genD1* gene is essential and acts exclusively in the conversion of **6** to **4**, consistent with a specific role in C-methylation at the C-4″ position. It appears likely that in the disruption mutant previously studied (Kim et al., 2008), there was a deleterious polar effect on the downstream *genS2* gene, which would account for the lack of accumulation of **6** in that study.

Purified Recombinant GenD2, GenS2, and GenN Together Catalyze Successive 3″-Oxidation, Transamination, and *N*-Methylation of Gentamicin A2 to Form Gentamicin A

To obtain direct evidence for the catalytic roles of GenD2, GenS2, and GenN, we turned to in vitro reconstitution experiments, starting with gentamicin A2 (**3**). This substrate was purified from fermentation extracts of the ΔgenS2ΔgenK strain of *M. echinospora* (Figure 2F), and its identity was confirmed by LC-HRMS. GenD2, GenS2, and GenN were each expressed in *Escherichia coli* as soluble N-His_6-tagged proteins and purified to near homogeneity (Figure S4A). The molecular weights of the proteins as analyzed by LC-MS were in agreement with the calculated values assuming loss of the N-terminal methionine: 38,533 Da for GenD2 (calculated 38,539 Da); 48,820 Da for GenS2 (calculated 48,826.5 Da); and 37,218 Da for GenN (calculated 37,221.8 Da), respectively. Adventitious *N*-gluconyl modification (+178 Da) (Geoghegan et al., 1999) was also seen with these proteins.

First, the predicted dehydrogenase GenD2 was incubated with **3**, and either NAD^+ or $NADP^+$ as redox cofactor, at 30°C. Samples were analyzed using LC-ESI-HRMS after 10 min, 60 min, 90 min, and overnight incubation. However, no DOA2 (**8**) was detected, even after overnight incubation. Addition of the aminotransferase GenS2, and L-glutamate as amino donor, also failed to reveal any of the expected DAA2 (**9**). In contrast, when all three enzymes (GenD2, GenS2, and GenN) were present, as well as NAD^+, L-glutamate, and SAM (as a methyl donor), essentially complete (>96%) conversion of **3** (Figure 3A) to **6** (Figure 3B) was achieved within 10 min at 30°C. The identity of the product was confirmed by LC-ESI-HRMS analysis, and comparison with authentic **6**: $[M+H]^+$ *m/z* = 469.2498 (−1.28 ppm) and $[M + Na]^+$ *m/z* = 491.2319 (−1.02 ppm) (Figure 3B). Reactions lacking any one of the three enzymes did not yield any product. These results strongly support the hypothesis that the 3″-modifications on **3** leading to **6** formation are indeed catalyzed by the coupled activities of GenD2, GenS2, and GenN. It is reasonable to speculate that the activities of GenD2 and GenS2 may be inhibited by very low levels of their products and that subsequent 3″-methylation by GenN alleviates this product inhibition.

The Aminoglycosides Kanamycin B and Tobramycin Are Deaminated by GenS2 and GenD2 and Methylated by GenN

To help confirm the respective roles of GenD2 and GenS2, kanamycin B (**11**) and tobramycin (**12**) were used. These aminoglycoside antibiotics are close structural analogs of DAA2 (**9**), and both possess an unmethylated 3″-amino group, so they could be tested as surrogate substrates for 3″-deamination, the reverse of the reaction normally catalyzed by GenS2/GenD2. In these assays, using 2-oxoglutarate as the amino acceptor, LC-ESI-HRMS analysis showed that GenS2 catalyzed low-level deamination of both compounds. The change could be localized to the kanosamine ring, consistent with the production, respectively, of trace amounts of 3″-deamino-3″-oxo-kanamycin B (**13**; $[M + H]^+$ *m/z* 483.2287, $[M + Na]^+$ *m/z* 505.2113) (Figure S5B) and 3″-deamino-3″-oxo-tobramycin (**14**; $[M + H]^+$ *m/z* 467.2342) (Figure S5F). In contrast, in the presence of GenS2 and GenD2 (and either NADH or NADPH) essentially quantitative yields were obtained of, respectively, 3″-deamino-3″-hydroxy-kanamycin B (**15**; $[M + H]^+$ *m/z* 485.2443, $[M + Na]^+$ *m/z* 507.2263)

Figure 2. Production of Gentamicins by *Micromonospora echinospora* Mutants
LC-ESI-HRMS total ion current traces of (A) gentamicin standard; and of mutant fermentation culture extracts from (B) wild-type; (C) ΔgenD2 mutant; (D) ΔgenS2 mutant; (E) ΔgenD2ΔgenK mutant; (F) ΔgenS2ΔgenK mutant; (G) ΔgenD2::*genD2* mutant; (H) ΔgenS2::*genS2* mutant; (I) ΔgenD2ΔgenK::*genK* mutant; (J) ΔgenS2ΔgenK::*genK* mutant; (K) ΔgenD1 mutant; (L) ΔgenN mutant; (M) ΔgenD1::*genD1* mutant; (N) ΔgenN::*genN* mutant. For the structure of metabolites, see Figure 1.

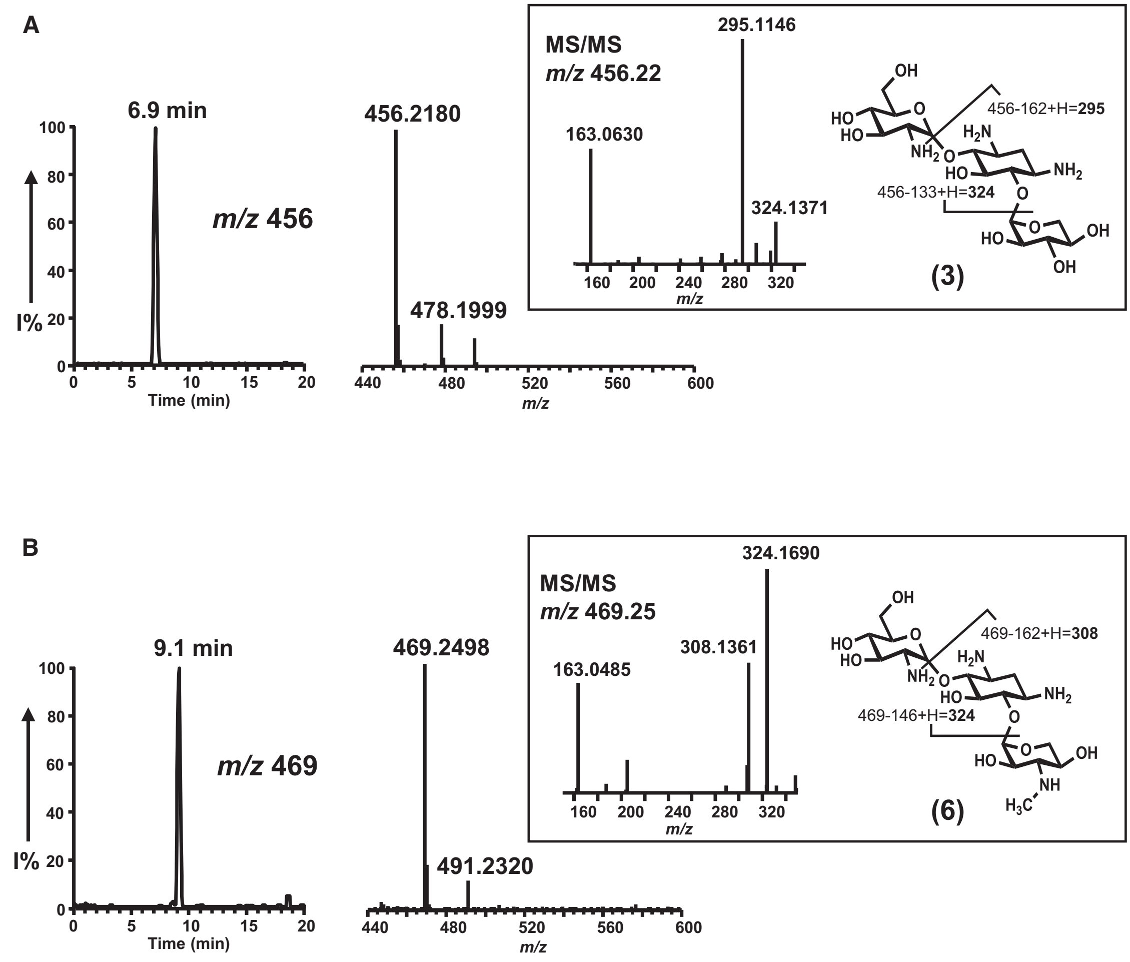

Figure 3. Enzymatic One-Pot Conversion of Gentamicin A2 to Gentamicin A

LC-ESI-HRMS selective ion monitoring was carried out on (A) $[M + H]^+$ (*m/z* 456) and $[M + Na]^+$ (*m/z* 478) ions of gentamicin A2 (**3**); (B) $[M + H]^+$ (*m/z* 469) and $[M + Na]^+$ (*m/z* 491) ions of gentamicin A (**6**), produced by the coupled action of GenD2, GenS2, and GenN on gentamicin A2 (**3**). MS/MS fragments from the $[M + H]^+$ (*m/z* 456) and $[M + H]^+$ (*m/z* 469) ions are shown as insets.

(Figure S5C) and 3″-deamino-3″-hydroxy-tobramycin (**16**; $[M + H]^+$ *m/z* 469.2496, $[M + Na]^+$ *m/z* 491.2314) (Figure S5G).

11 and **12** were excellent substrates for SAM-dependent methylation by GenN, the reaction being complete after 1 hr at 30°C. For kanamycin B GenN showed k_{cat} = 1.93 ± 0.1 s^{-1} and K_m = 34 ± 3.8 μM (see Supplemental Experimental Procedures for assay details). LC-ESI-HRMS analysis confirmed that methylation in both cases occurred specifically on the kanosamine ring, consistent with the formation, respectively, of 3″-*N*-methyl-kanamycin B (**17**; $[M + H]^+$ *m/z* 498.2761 (−1.81 ppm), $[M + Na]^+$ *m/z* 520.2582 (−1.35 ppm)) (Figure S5D) and 3″-*N*-methyltobramycin (**18**; $[M + H]^+$ *m/z* 482.2814 (−1.45 ppm), $[M + Na]^+$ *m/z* 504.2633 (−1.39 ppm)) (Figure S5H). In contrast, GenN had no effect on **6**, **4**, or **5**, all of which are gentamicin intermediates already methylated at the 3″-N position. These results further confirm the 3″-oxidoreductase activity of GenD2, the 3″-aminotransferase activity of GenS2, and the 3″-*N*-methylation activity of GenN. Purified **15**, when used as substrate for the forward reaction, gave neither detectable **13** with GenS2 alone nor detectable **11** with GenS2 and GenD2 together. However, **15** was smoothly converted by GenS2, GenD2, and GenN into **17**.

The Radical SAM Methyltransferase GenD1 Catalyzes Cobalamin-Dependent Methylation of Gentamicin A to Produce Gentamicin X2

The putative radical SAM enzyme GenD1 is one of two such intriguing enzymes in the gentamicin pathway, the other being GenK, which catalyzes C-methylation of gentamicin X2 (**4**) at the C-6′ position (Kim et al., 2013). Like GenK, a putative cobalamin-dependent radical SAM C-methyltransferase, and like Sis14 from the gene cluster for the closely related aminoglycoside sisomicin (**19**; Supplemental Experimental Procedures), GenD1 houses a sequence motif (Cx_4Cx_2C) highly similar to the conserved Cx_3Cx_2C binding site for the [4Fe-4S] iron-sulfur cluster found in the radical SAM superfamily (Kim et al., 2013). The iron-sulfur cluster would mediate homolysis of SAM, triggering formation of a gentamicin substrate radical which in turn recruits a methyl group from methylcobalamin. Important preliminary evidence has recently been presented for the in vitro activity of GenK,

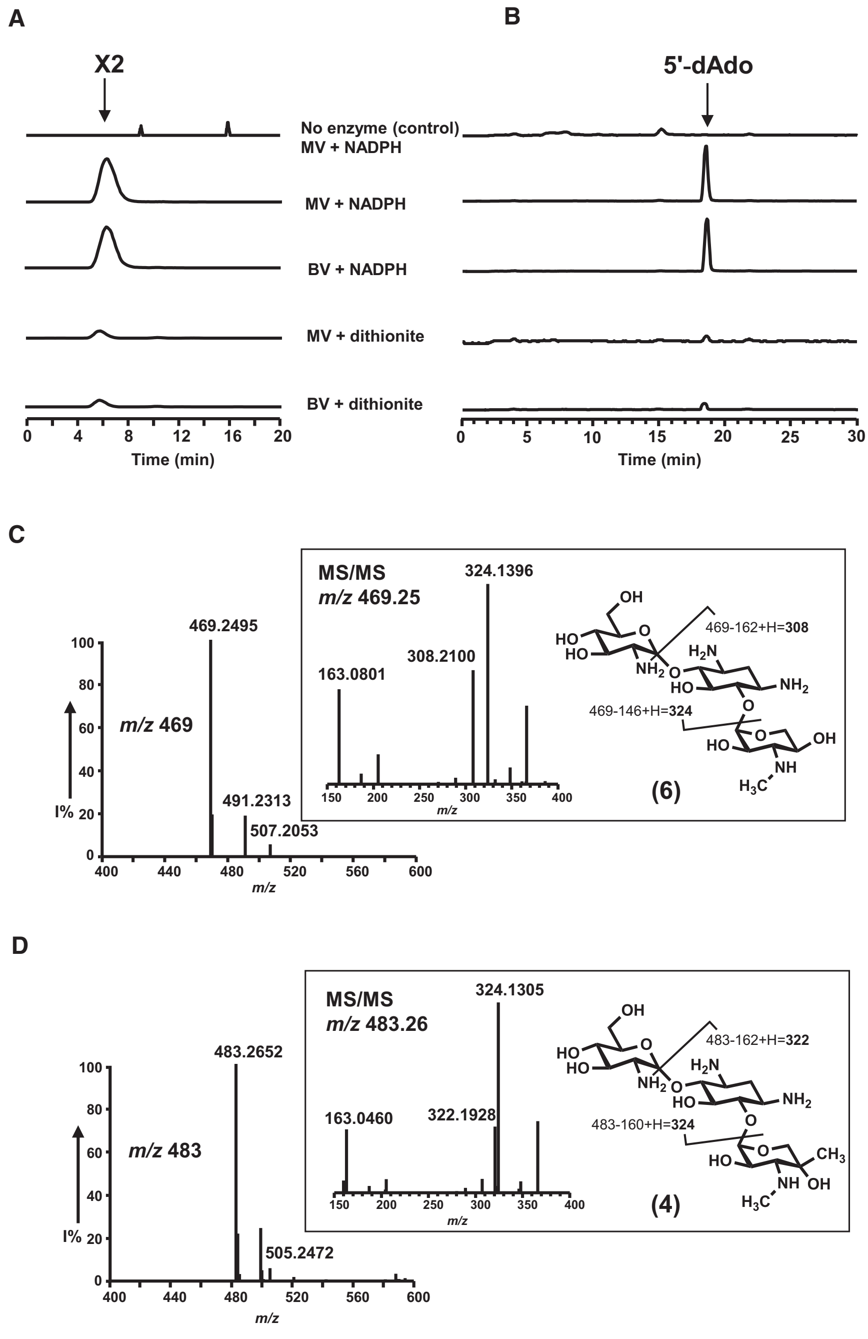

Figure 4. C-Methylation of Gentamicin A to Gentamicin X2 Catalyzed by GenD1

LC-ESI-HRMS selective ion monitoring was carried out on (A) $[M + H]^+$ *m/z* 483 of the product of GenD1-catalyzed methylation of gentamicin A (**6**); (B) $[M + H]^+$ *m/z* 252 of coproduced 5′-dAdo (**20**); (C) MS and MS/MS spectra of gentamicin A (**6**); (D) MS and MS/MS spectra of gentamicin X2 (**4**). Different high-performance liquid chromatography conditions were used for detection of gentamicin X2 (**4**) and 5′-dAdo (**20**), respectively, as described in Supplemental Experimental Procedures. 5′-dAdo, 5′-deoxyadenosine; BV, benzyl viologen; MV, methyl viologen.

including the involvement of cobalamin (Kim et al., 2013). GenD1 was accordingly expressed in *E. coli* as a soluble N-His$_6$-tagged protein and purified to near homogeneity (Figure S5A). The molecular weight of purified recombinant GenD1 as determined by LC-MS analysis was 77,410 Da, consistent with the calculated mass (77,417.5 Da) of the protein lacking the first methionine. Affinity purification, buffer exchange to remove imidazole, and reconstitution of the iron-sulfur cluster were carried out in an anaerobic chamber to minimize oxidative damage. Purified GenD1 had a brownish color. After reconstitution of the iron-sulfur cluster in GenD1 using ferrous ammonium sulfate and sodium sulfide, the protein solution assumed a gray-black color, as reported for other radical SAM-dependent methyltransferases, and showed a UV-visible absorbance peak centered on 415 nm (Figure S4B) as expected for a protein containing an iron-sulfur cluster (Duin et al., 1997; Pierrel et al., 2002; Kim et al., 2013). The color of the protein faded gradually within hours upon exposure to air.

Freshly purified and reconstituted GenD1 was assayed for its ability to catalyze C-methylation of **6** at the C-4″ position to yield **4**, using conditions previously found successful for GenK (Kim et al., 2013). Incubations (12 hr) were carried out in an anaerobic chamber at 30°C and the reaction mixtures were analyzed by using LC-ESI-HRMS. Reaction mixtures contained commercially available **6** (0.4 mM), SAM (4 mM), dithiothreitol (DTT) (10 mM) and methylcobalamin (1 mM) in 50 mM Tris-HCl buffer (pH 8.0). The reaction was initiated by addition of GenD1 (50 μM). With sodium dithionite (4 mM) as the source of electrons, and with either methyl viologen (MV) or benzyl viologen (BV) (each at 1 mM) present to mediate reductive activation of the iron-sulfur center, only modest (4%–5%) conversion of **6** to a methylated product was seen. However, conversion was increased to 84%–88% when the source of electrons was NADPH (4 mM) (Figure 4A). The methylated product had a mass consistent with that of **4**: $[M + H]^+$ *m/z* 483.2650 (−2.28 ppm); $[M + Na]^+$ *m/z* 505.2468 (−2.37 ppm). Its MS-MS fragmentation pattern, identical to that of authentic **4** (Figure 4B), confirmed that the methylation had occurred as expected on the C-4″ position of **6**. As found for GenK, methylcobalamin could be replaced by hydroxocobalamin (14%–18% conversion), suggesting that methylcobalamin can be regenerated by methyltransfer from SAM during catalysis. **4** was not formed in the absence of either cobalamin or viologen. Omission of the reconstitution step, or carrying out assays under aerobic conditions, gave little or no activity. The formation of 5′-deoxyadenosine (**20**, 5′-dAdo**)** as a reaction product

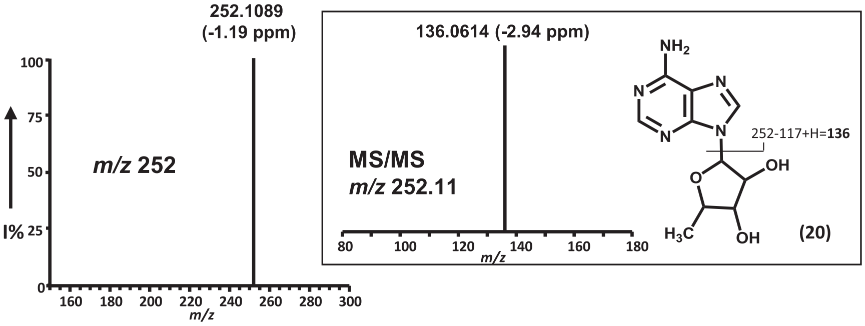

Figure 5. Production of 5′-Deoxyadenosine during GenD1-Catalyzed Methylation of Gentamicin A

LC-ESI-HRMS selective ion monitoring and MS/MS fragmentation of 5′-deoxyadenosine (5′-dAdo) (**20**), $[M + H]^+$ *m/z* 252.

was also confirmed by LC-ESI-HRMS ($[M+H]^+$ *m/z* 252.1089 (−1.19 ppm)) (Figures 4A and 5), suggesting a similar catalytic mechanism to that proposed for GenK, in which a 5′-dAdo radical generated via the reductive cleavage of SAM abstracts a hydrogen from the substrate, leading to the formation of a substrate radical (Kim et al., 2013).

DISCUSSION

The confident application of synthetic biology to reconfigure gene sets and thus redirect the course of an antibiotic biosynthetic pathway requires an excellent understanding of the underlying enzymology. For the biosynthetic pathway to the gentamicin C complex, an established drug to treat life-threatening infections caused by Gram-negative bacteria, even the task of assigning functions to individual gene products involved in the pathway is not trivial because the cluster contains multiple, mutually homologous genes for methyltransferases, oxidoreductases, and aminotransferases with potential for overlapping substrate specificities. There are numerous precedents from other aminoglycoside pathways for enzyme promiscuity or even dual function (Huang et al., 2007; Fan et al., 2008; Yokoyama et al., 2008; Park et al., 2011). From our analysis of the ΔgenD2, ΔgenS2, and ΔgenN mutants of *M. echinospora* it is clear that GenD2 (dehydrogenase), GenS2 (aminotransferase), and GenN (*N*-methyltransferase) uniquely govern the replacement of the C-3″-hydroxyl group in gentamicin A2 (**3**) by the methylamino group of gentamicin X2 (**4**). No other enzymes in *M. echinospora* were able to take over these roles under the fermentation conditions used, and these enzymes are apparently not required elsewhere in the pathway. The appearance in these mutants of the shunt metabolite gentamicin A2e (**7**) is readily explained by the action of the methyltransferase GenK on the earlier precursors gentamicin A2 (**3**) and gentamicin A (**6**) in the absence of its normal substrate.

The selective production of gentamicin A2 (**3**) from the ΔgenD2ΔgenK mutant provided the substrate to study the recombinant enzymes in vitro. The in vitro work, in turn, confirmed the conclusions from the initial analysis of specific gene knockouts but importantly also revealed that the GenD2/GenS2-catalyzed dehydrogenation/amination only proceeds when the gentamicin A (**6**) product is removed (by GenN-catalyzed methylation) as fast as it is formed. The simplest explanation for this observation is the relief of product inhibition, a neat way to avoid unwanted buildup of reactive pathway intermediates. Indirect confirmation of the likely nature of those intermediates between gentamicin A2 (**3**) and gentamicin A (**6**) could nevertheless be obtained by studying the reactions in reverse, using as substrate analogs of **6** the structurally related aminoglycosides kanamycin B (**11**) and tobramycin (**12**), which each have a free 3″-amino group. GenS2 alone did not catalyze transamination of either substrate but each was efficiently converted into the respective 3″-hydroxy-derivative by the joint action of GenS2 and GenD2. The 3″-hydroxy-kanamycin B (**15**), when used as a substrate for the forward reaction, behaved exactly like gentamicin A2 (**3**): no reaction with GenD2 and GenS2 unless all three enzymes (GenD2, GenS2, and GenN) were present, strongly suggesting that the presence of GenN relieves product inhibition by **13** and **11**.

It has been previously suggested (Kim et al., 2008) that GenD1 catalyzes the *N*-methylation at C-3″ of gentamicin A (**6**), but it is clear from our present work that GenN governs this step. Rather, GenD1 can be confidently assigned, on the basis of both in vivo gene disruption and direct in vitro assay, as the C-methyltransferase that transforms gentamicin A (**6**) into gentamicin X2 (**4**). After GenK (Kim et al., 2013), GenD1 is the second representative of this mechanistically intriguing class of cobalamin-dependent radical SAM methyltransferases to be characterized in the gentamicin pathway. Both these enzymes activate and achieve substitution at unactivated *sp3* C centers. Unlike GenK, which has required refolding from inclusion bodies after heterologous expression (Kim et al., 2013), GenD1 is expressed in *E. coli* as a soluble protein in excellent yield, which should be helpful in future mechanistic study of this enzyme.

SIGNIFICANCE

Aminoglycosides, mainly produced by actinobacteria, constitute a vital clinical asset. Gentamicin in particular is of continuing interest because of its remarkable potency in treating systemic Gram-negative infections, and yet because of the significant known toxicity of the gentamicin complex it requires constant and expensive individual monitoring of patients. The perspective of a safer and less expensive gentamicin through administration of a single component, or using a single component for semisynthesis of a novel derivative, is therefore very attractive. The work we report here has used complementary in vivo and in vitro approaches to identify the four key enzymes that lead from the first-formed pseudotrisaccharide to gentamicin X2, the most advanced common precursor of all the components of gentamicin C complex. It has confirmed the activity of soluble recombinant GenD1 as a cobalamin-dependent radical SAM methyltransferase in this pathway, paving the way for detailed mechanistic study of this intriguing enzyme; and, by more closely defining the

molecular enzymology of the pathway in *M. echinospora*, has brought closer the goal of assembling a defined set of enzymes to deliver single gentamicin C components.

EXPERIMENTAL PROCEDURES

Bacterial Strains and Plasmids, Chemicals, and Culture Conditions

E. coli strains NovaBlue and BL21(DE3) (Novagen) were used as cloning and expression hosts, respectively. For routine cloning *E. coli* strains were maintained in 2× TY medium (tryptone 1.6%, yeast extract 1%, NaCl 0.5%) at 37°C with appropriate antibiotic selection at the indicated final concentrations: ampicillin (100 μg/ml), apramycin (50 μg/ml), kanamycin (25 μg/ml), or chloramphenicol (25 μg/ml). For *E. coli* protein expression, Luria-Bertani (LB) medium (1% tryptone, 0.5% yeast extract, 1% NaCl) was used at 37°C with appropriate antibiotic selection at the indicated final concentrations: tetracycline (15 μg/ml) for NovaBlue cells, kanamycin (50 μg/ml) for cells harboring the recombinant pET28a(+) plasmids, and carbenicillin (100 μg/ml) for plasmid pDB1282, containing essential genes for biosynthesis of the iron-sulfur cluster (Zheng et al., 1998). Ferrous ammonium sulfate, LB medium, imidazole, NaCl, and Tris base were purchased from Fisher Scientific. Restriction endonucleases, *Pfu* DNA polymerases, and T4 DNA ligase were from Fermentas (Thermo Scientific). Oligonucleotide primers were synthesized by Invitrogen Life Technologies. Gentamicin A (**6**), gentamicin X2 (**4**), and kanamycin B (**11**) were products from Toku-E. G418 used for feeding studies was from Gibco. Amino acids, hydroxocobalamine, methylcobalamine (MeCbl), MV, BV, SAM, sodium dithionite, sodium sulfide, and tobramycin (**12**) were obtained from Sigma-Aldrich.

M. echinospora ATCC15835 wild-type and mutants were grown on ATCC 172 medium (glucose 1%, yeast extract 0.5%, soluble starch 2%, N-Z amine 0.5%, $CaCO_3$ 0.2%) at 28°C for genomic DNA isolation and cultivation. For aminoglycoside production, the seed culture was shaken at 220 rpm in liquid ATCC 172 medium at 28°C for 2 days and then used to initiate the fermentation culture (5% inoculum) in fermentation medium (Soybean powder 2.0%, peptone 0.1%, glucose 0.3%, $(NH_4)_2SO_4$ 0.03%, $CaCO_3$ 0.3%, KNO_3 0.03%, $CoCl_2$ 5 ppm) shaken at 220 rpm and at 28°C for 5 days. For feeding experiments, filter-sterilized compounds (150 μg/ml) were added to the fermentation medium before inoculation.

Isolation and Analysis of Gentamicin and Intermediates

The fermentation broth was adjusted to pH 2.0 with concentrated HCl. The cultures were agitated for 2 hr on a laboratory rocker and then centrifuged at 5000 *g* for 10 min at 4°C. Each supernatant was applied to a column of 1.5 g DOWEX 50WX8-200 ion-exchange resin preconditioned with 50 ml acetonitrile followed by 50 ml × 4 distilled-deionized water. The column was washed with 15 ml of water and then aminoglycosides were eluted with 15 ml of 1 M ammonium hydroxide. The eluate was freeze-dried, the residue was dissolved in 1 ml of water, and a sample was subjected to high-performance liquid chromatography-HRMS analysis.

Construction of Gene Disruption Plasmids

For in-frame deletion, DNA fragments flanking each target gene were amplified from the genomic DNA of *M. echinospora* ATCC15835 using Phusion DNA polymerase (New England Biolabs). The PCR products were each cloned into pUC18, then cut out and cloned together into the *Streptomyces-E. coli* shuttle vector pYH7 (Sun et al., 2008) to obtain the following gene disruption plasmids: pWHU6 (for ΔgenD2), pWHU21 (for ΔgenS2), pYH289 (for ΔgenN), and pYH287 (for ΔgenD1). For construction of the ΔgenD2ΔgenK and ΔgenS2ΔgenK double mutants, pWHU1 (Guo et al., 2014) was employed for *genK* in-frame deletion. All plasmids were verified by sequencing.

Construction of Gene Complementation Plasmids

Complementation plasmids were prepared by cloning *genD2*, *genS2*, *genN*, *genD1*, and *genK*, respectively, into pWHU77 (a plasmid derived from pIB139 [Wilkinson et al., 2002; Del Vecchio et al., 2003] with the apramycin resistance gene replaced by a thiostrepton resistance gene) under the control of the P*ermE** promoter (Figure S1). The PCR products were inserted into pWHU77 between the NdeI and EcoRI sites to generate pWHU184, pWHU115, pWHU68, pWHU66, and pWHU67. After sequence confirmation, these plasmids were introduced individually into ΔgenD2 (pWHU184), ΔgenS2 (pWHU115), ΔgenN (pWHU68), ΔgenD1 (pWHU66), ΔgenD2ΔgenK (pWHU67), and ΔgenS2ΔgenK (pWHU67) by conjugation. Complemented exconjugants were verified based on thiostrepton resistance and confirmed by PCR (Figure S1).

Targeted In-Frame Gene Deletion

To create individual in-frame deletion mutants of *genD2*, *genS2*, *genN*, and *genD1*, the corresponding plasmid pWHU6, pWHU21, pYH289, and pYH287 was introduced into the wild-type strain by conjugation from *E. coli* ET12567/pUZ8002 (MacNeil et al., 1992) on ABB medium (soytone 0.5%, soluble starch 0.5%, $CaCO_3$ 0.3%, MOPS 0.21%, $FeSO_4$ 0.0012%, thiamine-HCl 0.001%, agar 3%) with addition of 10 mM $MgCl_2$ solution. After 10 hr incubation at 28°C, the exconjugants were selected with nalidixic acid (12.5 μg/ml) and apramycin (12.5 μg/ml). Exconjugants were transferred onto ABB medium containing nalidixic acid (25 μg/ml) and apramycin (25 μg/ml). To promote a second crossover, these mutants were propagated on A medium (soluble starch 1%, corn steep powder 0.25%, yeast extract 0.3%, $CaCO_3$ 0.3%, $FeSO_4$ 0.0012%, agar 3% [pH 7.0], adjusted with KOH) with addition of 10 mM $MgCl_2$ solution. To select the recombinant progeny by their apramycin-sensitive phenotype, single colonies on A plates were patched in parallel onto antibiotic (25 μg/ml apramycin)-containing A medium and antibiotic-free A medium. The desired in-frame deletion mutants were identified by PCR using the checking primers and further confirmed by Southern blot analysis (Figures S1A, S1B, S1E, and S1F). Double in-frame deletion mutants, ΔgenD2ΔgenK and ΔgenS2ΔgenK, were prepared using the same protocol with ΔgenD2 and plasmids pWHU1 (Figures S1C and S1D).

Gene Complementation of the ΔgenD2, ΔgenS2, ΔgenN, ΔgenD1, ΔgenD2ΔgenK, and ΔgenS2ΔgenK Mutants

The complementation plasmids were introduced individually into ΔgenD2 (pWHU184), ΔgenS2 (pWHU115), ΔgenN (pWHU68), ΔgenD1 (pWHU66), ΔgenD2ΔgenK (pWHU67), and ΔgenS2ΔgenK (pWHU67) by conjugation. Complemented exconjugants were identified based on thiostrepton resistance and confirmed by PCR (Figures S1G–S1L).

Cloning of *genD1*, *genD2*, *genN*, and *genS2* Genes for Expression in *E. coli*

The *genD2*, *genS2*, *genN*, and *genD1* genes were each amplified from the genomic DNA of *M. echinospora* ATCC15835 by PCR using *Pfu* DNA polymerase with 25 cycles of denaturing at 94°C for 1 min, annealing at 60°C for 1 min, and extension at 72°C for 2 min plus a final extension at 72°C for 10 min. The PCR products were digested with appropriate restriction enzymes, purified by gel extraction (Fermentas), and inserted into a pET28a(+) plasmid. The resulting constructs were verified by DNA sequencing.

Overexpression and Purification of Recombinant Proteins

E. coli BL21(DE3) cells bearing the recombinant plasmids were cultured in LB broth containing kanamycin (50 μg/ml) at 37°C until the cell density reached 0.5–1.0 at 600 nm. Overexpression of the proteins was induced by isopropylthiogalactoside (IPTG) (0.1 mM) at 20°C with shaking at 180 rpm overnight. For overexpression of GenD1, *E. coli* BL21(DE3) cells harboring both *genD1* gene in pET28a(+) plasmid and pDB1282 plasmid containing the iron-sulfur cluster biosynthetic genes (Zheng et al., 1998) were incubated in 1.5 l of LB broth containing kanamycin and carbenicillin in a 2 l flask at 37°C, 150 rpm until the cell density reached $A_{600} = 0.8 - 1.0$. The iron-sulfur cluster protein expression was induced with 20 mM L-(+)-arabinose and the culture incubated for a further 45–60 min. The culture was then cooled to room temperature before addition of 0.2 mM IPTG to induce GenD1 protein expression at 20°C, 150 rpm overnight; 2 mM Fe(II)SO_4 was also added at this point. Cells were harvested by centrifugation and resuspended in Binding Buffer (0.5 M NaCl, 20 mM Tris-HCl [pH 7.9]). The Binding Buffer for GenD1 resuspension was purged with N_2 for at least 30 min before use. The recombinant protein was released by sonication for 4 min using a 2 s on/6 s off cycle and the recombinant protein in the clarified cell lysate was purified using Co^{2+} or Ni^{2+} ion-charged His-Bind metal chelating resin (Novagen) according to the manufacturer's instructions. Imidazole in the eluted GenD2, GenS2, and GenN solutions was removed by buffer exchange using Amicon Ultra centrifugal filters (Millipore). The purification

procedure for GenD1 from the His-Bind purification and onward was carried out in a DAB-10S anaerobic chamber (Saffron Scientific). Imidazole was removed from the purified GenD1 protein solution using a PD10 column (GE Healthcare) with a buffer containing 0.25 M NaCl and 20 mM Tris-HCl (pH 7.9). Anaerobic buffers were prepared outside the anaerobic chamber and autoclaved. Autoclaved buffers were then purged with nitrogen for at least 30 min with stirring before being transferred to the anaerobic chamber, and allowed to stir uncapped at least overnight. Chemical solutions to be stored at −20°C were prepared within the anaerobic chamber and transferred immediately to −20°C after removal from the anaerobic chamber. Chemical solutions freshly prepared on the day of use were dissolved in anaerobic water inside the anaerobic chamber. Tips, tubes, and other consumables were autoclaved and stored in the anaerobic chamber.

The purified proteins were stored at −20°C in a buffer containing 250 mM NaCl, 10 mM Tris-HCl (pH 7.9), and 33% glycerol. The identities of the purified recombinant proteins were confirmed by SDS-PAGE, UV-visible absorbance analysis (Cary 100 Bio UV-visible spectrophotometer), and LC-ESI-MS (ThermoFinnigan). Protein concentrations were determined using Bradford protein dye reagent (Sigma).

Reconstitution of the Iron-Sulfur Cluster in GenD1

The reconstitution of the iron-sulfur cluster in GenD1 protein was conducted under strictly anaerobic conditions immediately after elution from PD-10 columns. The average temperature in the glove box was 30°C. The protein solution (2.5 ml) was incubated with 5 mM DTT for 15 min followed by addition of 1 mM $Fe(II)(NH_4)_2(SO_4)_2$ and 1 mM Na_2S, both were freshly prepared, yielding a translucent, blackish solution. The solution was left at room temperature for 1 hr, and was then exchanged into a buffer containing 0.25 M NaCl and 20 mM Tris-HCl (pH 7.9) using a PD-10 column. The eluted protein solution remained grayish black, indicating a successful reconstitution. A portion of the reconstituted protein was transferred to a quartz cuvette with a lid and sealed with Parafilm, and was immediately subjected to UV-vis scanning. The reconstituted protein was stored at −80°C, in Eppendorf tubes wrapped in Parafilm, after addition of glycerol to 33% (v/v).

Activity Assays of GenD2, GenS2, and GenN

Gentamicin A2 (**3**) purified from the ΔgenS2ΔgenK mutant of *M. echinospora* ATCC15835 (see Supplemental Experimental Procedures, Method S1, for the protocol of purification) was used as a substrate in assays testing the activity of GenD2 as a 3″-dehydrogenase, GenS2 as a 3″-transaminase, and GenN as a 3″-*N*-methyltransferase. A typical reaction (100 μl) contained substrate (400 μM), NAD^+ or $NADP^+$ (2.5 mM), L-glutamate (1 mM, when GenS2 was present), SAM (2 mM, when GenN was present), and purified enzyme(s) (30 μM each) in Tris-HCl buffer (50 mM, pH 7.5). To test the deamination activity of GenS2 and the subsequent ketoreductase activity of GenD2 (the reverse reactions), kanamycin B (**11**) and tobramycin (**12**) were used as substrates. A typical reaction mixture consisted of substrate (400 μM), NADH or NADPH (2.5 mM), 2-oxoglutarate (1 mM, when GenS2 was present), and purified enzyme(s) (30 μM) in Tris-HCl buffer (50 mM, pH 7.5). Kanamycin and tobramycin were used as substrate analogs to test the putative 3″-*N*-methyltransferase activity of GenN. A typical reaction mixture contained substrate (400 μM), SAM (2 mM), and purified GenN (30 μM) in Tris-HCl buffer (50 mM, pH 7.5). Reaction mixtures were incubated at 30°C and quenched by addition of chloroform (100 μl) followed by vigorous vortexing. Precipitated protein was removed by centrifugation. Ten to twenty microliters of the aqueous supernatant were analyzed by either LC-ESI-MS or LC-ESI-HRMS.

GenD1 Assay

Assays were carried out in an anaerobic chamber (~30°C) overnight. Standard reaction mixtures (50 μl) contained 1 mM MeCbl, 10 mM DTT, 4 mM SAM, 100 μM purified reconstituted GenD1, 400 μM substrate, 4 mM NADPH, and 1 mM MV in 50 mM Tris-HCl (pH 8). Alternative reducing agents tested were combinations of 4 mM NADPH or $Na_2S_2O_4$ and 1 mM MV or BV. After incubation, assays were removed from the anaerobic chamber and protein was precipitated by addition of 50 μl of chloroform. The assays were vortexed and then centrifuged at 13,000 rpm for 5 min. Ten to twenty microliters of the aqueous supernatant were analyzed by either LC-ESI-MS or LC-ESI-HRMS.

LC-ESI-MS Analyses

Conditions for LC-ESI-MS analyses were as described in Supplemental Experimental Procedures.

SUPPLEMENTAL INFORMATION

Supplemental Information including one table, five figures, and Supplemental Experimental Procedures can be found with this article online at http://dx.doi.org/10.1016/j.chembiol.2014.12.012.

AUTHOR CONTRIBUTIONS

F.H., C. H., P.F.L., and Y.S. conceived the experiments; C. H., J.G., X.J., and X.D. constructed and analyzed mutants; F.H. and E.M. carried out in vitro analysis; C.H., F.H., E.M., J.G., Z.D., P.F.L., and Y.S. analyzed the results; and F.H., C. H., P.F.L., and Y.S. wrote the paper.

ACKNOWLEDGMENTS

This work was supported by a project grant from the Medical Research Council, UK (G1001687) to P.F.L.; and by the 973 and 863 programs from the Ministry of Science and Technology of China, National Science Foundation of China, and the Translational Medical Research Fund of Wuhan University School of Medicine to Y.S.; E.M. thanks the Gates Cambridge Trust for a scholarship. We also gratefully acknowledge Dr. Xinzhou Yang, South-Central University for Nationalities, for his assistance in separation of gentamicin A2. We thank Dr. Andrew Truman (John Innes Institute) for helpful discussions.

Received: September 22, 2014
Revised: December 3, 2014
Accepted: December 6, 2014
Published: January 29, 2015

REFERENCES

Aboshanab, K.M.A. (2005). Genetic Studies on the Biosynthesis of the Major Aminoglycoside Antibiotics: Isolation, Analysis and Comparison of the Biosynthetic Gene Clusters for 12 Aminoglycoside Antibiotics, PhD Thesis. (Wuppertal: Bergische Universität Wuppertal).

Bockenhauer, D., Hug, M.J., and Kleta, R. (2009). Cystic fibrosis, aminoglycoside treatment and acute renal failure: the not so gentle micin. Pediatr. Nephrol. *24*, 925–928.

Cuccarese, M.F., Singh, A., Amiji, M., and O'Doherty, G.A. (2013). A novel use of gentamicin in the ROS-mediated sensitization of NCI-H460 lung cancer cells to various anticancer agents. ACS Chem. Biol. *8*, 2771–2777.

Del Vecchio, F., Petkovic, H., Kendrew, S.G., Low, L., Wilkinson, B., Lill, R., Cortés, J., Rudd, B.A.M., Staunton, J., and Leadlay, P.F. (2003). Active-site residue, domain and module swaps in modular polyketide synthases. J. Ind. Microbiol. Biotechnol. *30*, 489–494.

Duin, E.C., Lafferty, M.E., Crouse, B.R., Allen, R.M., Sanyal, I., Flint, D.H., and Johnson, M.K. (1997). [2Fe-2S] to [4Fe-4S] cluster conversion in *Escherichia coli* biotin synthase. Biochemistry *36*, 11811–11820.

Fan, Q.Z., Huang, F.L., Leadlay, P.F., and Spencer, J.B. (2008). The neomycin biosynthetic gene cluster of *Streptomyces fradiae* NCIMB 8233: genetic and biochemical evidence for the roles of two glycosyltransferases and a deacetylase. Org. Biomol. Chem. *6*, 3306–3314.

Geoghegan, K.F., Dixon, H.B., Rosner, P.J., Hoth, L.R., Lanzetti, A.J., Borzilleri, K.A., Marr, E.S., Pezzullo, L.H., Martin, L.B., LeMotte, P.K., et al. (1999). Spontaneous alpha-N-6-phosphogluconoylation of a "His tag" in *Escherichia coli*: the cause of extra mass of 258 or 178 Da in fusion proteins. Anal. Biochem. *26*, 169–184.

Guo, J., Huang, F., Huang, C., Duan, X., Jian, X., Leeper, F., Deng, Z., Leadlay, P.F., and Sun, Y. (2014). Specificity and promiscuity at the branch point in gentamicin biosynthesis. Chem. Biol. *21*, 608–618.

Hainrichson, M., Nudelman, I., and Baasov, T. (2008). Designer aminoglycosides: the race to develop improved antibiotics and compounds for the treatment of human genetic diseases. Org. Biomol. Chem. *6*, 227–239.

Hong, W.R., and Yan, L.B. (2012). Identification of *gntK*, a gene required for the methylation of purpurosamine C-6′ in gentamicin biosynthesis. J. Gen. Appl. Microbiol. *58*, 349–356.

Hong, W.R., Ge, M., Zeng, Z.H., Zhu, L., Luo, M.Y., Shao, L., and Chen, D.J. (2009). Molecular cloning and sequence analysis of the sisomicin biosynthetic gene cluster from *Micromonospora inyoensis*. Biotechnol. Lett. *31*, 449–455.

Houghton, J.L., Green, K.D., Chen, W., and Garneau-Tsodikova, S. (2010). The future of aminoglycosides: the end or renaissance? Chembiochem *11*, 880–902.

Huang, F.L., Haydock, S.F., Mironenko, T., Spiteller, D., Li, Y., and Spencer, J.B. (2005). The neomycin biosynthetic gene cluster of *Streptomyces fradiae* NCIMB8233: characterisation of an aminotransferase involved in the formation of 2-deoxystreptamine. Org. Biomol. Chem. *3*, 1410–1419.

Huang, F.L., Spiteller, D., Koorbanally, N.A., Li, Y., Llewellyn, N.M., and Spencer, J.B. (2007). Elaboration of neosamine rings in the biosynthesis of neomycin and butirosin. Chembiochem *8*, 283–288.

Karki, S., Kim, J.Y., Park, S.H., and Kwon, H.J. (2012). Gene inactivation study on *gntK*, a putative C-methyltransferase gene in gentamicin biosynthesis from *Micromonospora echinospora*. J. Kor. Soc. Appl. Biol. Chem. *55*, 439–442.

Kase, H., Odakura, Y., and Nakayama, K. (1982a). Sagamicin and the related aminoglycosides: fermentation and biosynthesis I. Biosynthetic studies with the blocked mutants of *Micromonospora sagamiensis*. J. Antibiot. *35*, 1–9.

Kase, H., Shimura, G., Iida, T., and Nakayama, K. (1982b). Biotransformation of sisomicin and verdamicin by *Micromonospora sagamiensis*. Agric. Biol. Chem. *46*, 515–522.

Kharel, M.K., Basnet, D.B., Lee, H.C., Liou, K., Woo, S.W., Kim, B.G., and Sohng, J.K. (2004a). Isolation and characterization of the tobramycin biosynthetic gene cluster from *Streptomyces tenebrarius*. FEMS Microbiol. Lett. *230*, 185–190.

Kharel, M.K., Basnet, D.B., Lee, H.C., Liou, K., Moon, Y.H., Kim, J.J., Woo, J.S., and Sohng, J.K. (2004b). Molecular cloning and characterization of a 2-deoxystreptamine biosynthetic gene cluster in gentamicin-producing *Micromonospora echinospora* ATCC15835. Mol. Cells *18*, 71–78.

Kharel, M.K., Subba, B., Basnet, D.B., Woo, J.S., Lee, H.C., Liou, K., and Sohng, J.K. (2004c). A gene cluster for biosynthesis of kanamycin from *Streptomyces kanamyceticus*: comparison with gentamicin biosynthetic gene cluster. Arch. Biochem. Biophys. *429*, 204–214.

Kim, J.Y., Suh, J.W., Kang, S.H., Phan, T.H., Park, S.H., and Kwon, H.J. (2008). Gene inactivation study of *gntE* reveals its role in the first step of pseudotrisaccharide modifications in gentamicin biosynthesis. Biochem. Biophys. Res. Commun. *372*, 730–734.

Kim, H.J., McCarty, R.M., Ogasawara, Y., Liu, Y.N., Mansoorabadi, S.O., Levieux, J., and Liu, H.W. (2013). GenK-catalyzed C-6′ methylation in the biosynthesis of gentamicin: isolation and characterization of a cobalamin-dependent radical SAM enzyme. J. Am. Chem. Soc. *135*, 8093–8096.

Kobayashi, M., Sone, M., Umemura, M., Nabeshima, T., Nakashima, T., and Hellström, S. (2008). Comparisons of cochleotoxicity among three gentamicin compounds following intratympanic application. Acta Otolaryngol. *128*, 245–249.

Kudo, F., and Eguchi, T. (2009). Biosynthetic genes for aminoglycoside antibiotics. J. Antibiot. *62*, 471–481.

Kudo, F., Sucipto, H., and Eguchi, T. (2009). Enzymatic activity of a glycosyltransferase KanM2 encoded in the kanamycin biosynthetic gene cluster. J. Antibiot. *62*, 707–710.

Li, D., Li, H., Ni, X., Zhang, H., and Xia, H.Z. (2013). Construction of a gentamicin C1a-overproducing strain of *Micromonospora purpurea* by inactivation of the *gacD* gene. Microbiol. Res. *168*, 263–267.

Linde, L., and Kerem, B. (2008). Introducing sense into nonsense in treatments of human genetic diseases. Trends Genet. *24*, 552–563.

Llewellyn, N.M., and Spencer, J.B. (2006). Biosynthesis of 2-deoxystreptamine-containing aminoglycoside antibiotics. Nat. Prod. Rep. *23*, 864–874.

MacNeil, D.J., Occi, J.L., Gewain, K.M., MacNeil, T., Gibbons, P.H., Ruby, C.L., and Danis, S.J. (1992). Complex organization of the *Streptomyces avermitilis* genes encoding the avermectin polyketide synthase. Gene *115*, 119–125.

Ota, Y., Tamegai, H., Kudo, F., Kuriki, H., Koike-Takeshita, A., Eguchi, T., and Kakinuma, K. (2000). Butirosin biosynthetic gene cluster from *Bacillus circulans*. J. Antibiot. *53*, 1158–1167.

Park, J.W., Hong, J.S., Parajuli, N., Jung, W.S., Park, S.R., Lim, S.K., Sohng, J.K., and Yoon, Y.J. (2008). Genetic dissection of the biosynthetic route to gentamicin A2 by heterologous expression of its minimal gene set. Proc. Natl. Acad. Sci. USA *105*, 8399–8404.

Park, J.W., Park, S.R., Nepal, K.K., Han, A.R., Ban, Y.H., Yoo, Y.J., Kim, E.J., Kim, E.M., Kim, D., Sohng, J.K., and Yoon, Y.J. (2011). Discovery of parallel pathways of kanamycin biosynthesis allows antibiotic manipulation. Nat. Chem. Biol. *7*, 843–852.

Park, S.R., Park, J.W., Ban, Y.H., Sohng, J.K., and Yoon, Y.J. (2013). 2-deoxystreptamine-containing aminoglycoside antibiotics: recent advances in the characterization and manipulation of their biosynthetic pathways. Nat. Prod. Rep. *30*, 11–20.

Pierrel, F., Björk, G.R., Fontecave, M., and Atta, M. (2002). Enzymatic modification of tRNAs: MiaB is an iron-sulfur protein. J. Biol. Chem. *277*, 13367–13370.

Sandoval, R.M., Reilly, J.P., Running, W., Campos, S.B., Santos, J.R., Phillips, C.L., and Molitoris, B.A. (2006). A non-nephrotoxic gentamicin congener that retains antimicrobial efficacy. J. Am. Soc. Nephrol. *17*, 2697–2705.

Subba, B., Kahrel, M.K., Lee, H.C., Liou, K., Kim, B.G., and Sohng, J.K. (2005). The ribostamycin biosynthetic gene cluster in *Streptomyces ribosidificus*: comparison with butirosin biosynthesis. Mol. Cells *20*, 90–96.

Sun, Y.H., He, X.Y., Liang, J.D., Zhou, X.F., and Deng, Z.X. (2008). Analysis of functions in plasmid pHZ1358 influencing its genetic and structural stability in *Streptomyces lividans*1326. Appl. Microbiol. Biotechnol. *82*, 303–310.

Testa, R.T., and Tilley, B.C. (1975). Biotransformation, a new approach to aminoglycoside biosynthesis. I. Sisomicin. J. Antibiot. *28*, 573–579.

Testa, R.T., and Tilley, B.C. (1976). Biotransformation, a new approach to aminoglycoside biosynthesis. II. Gentamicin. J. Antibiot. *29*, 140–146.

Thibodeaux, C.J., Melancon, C.E., III, and Liu, H.W. (2008). Natural-product sugar biosynthesis and enzymatic glycodiversification. Angew. Chem. Int. Ed. Engl. *47*, 9814–9859.

Unwin, J., Standage, S., Alexander, D., Hosted, T., Jr., Horan, A.C., and Wellington, E.M. (2004). Gene cluster in *Micromonospora echinospora* ATCC15835 for the biosynthesis of the gentamicin C complex. J. Antibiot. *57*, 436–445.

Wilkinson, C.J., Hughes-Thomas, Z.A., Martin, C.J., Böhm, I., Mironenko, T., Deacon, M., Wheatcroft, M., Wirtz, G., Staunton, J., and Leadlay, P.F. (2002). Increasing the efficiency of heterologous promoters in actinomycetes. J. Mol. Microbiol. Biotechnol. *4*, 417–426.

Yokoyama, K., Yamamoto, Y., Kudo, F., and Eguchi, T. (2008). Involvement of two distinct N-acetylglucosaminyltransferases and a dual-function deacetylase in neomycin biosynthesis. Chembiochem *9*, 865–869.

Zheng, L., Cash, V.L., Flint, D.H., and Dean, D.R. (1998). Assembly of iron-sulfur clusters. Identification of an iscSUA-hscBA-fdx gene cluster from *Azotobacter vinelandii*. J. Biol. Chem. *273*, 13264–13272.

Chemistry & Biology, Volume *22*

Supplemental Information

Delineating the Biosynthesis of Gentamicin X2, the Common Precursor of the Gentamicin C Antibiotic Complex

Chuan Huang, Fanglu Huang, Eileen Moison, Junhong Guo, Xinyun Jian, Xiaobo Duan, Zixin Deng, Peter F. Leadlay, and Yuhui Sun

Supplemental Information

Inventory of Supplemental Information

Table S1. LC-ESI-HRMS analysis of *genD2-genS2-genN-genD1* knock-out mutants. Related to Figure 2.

Figure S1. In-frame deletion of *genD2*, *genS2*, *genN* and *genD1* in *M. echinospora* ATCC 15835, and complementation of ΔgenD2, ΔgenS2, ΔgenN, ΔgenD1, ΔgenD2ΔgenK and ΔgenS2ΔgenK. Related to Figure 2.

Figure S2. MS and MS/MS spectra of gentamicin-related intermediates isolated from mutants of *M. echinospora*. Related to Figure 2.

Figure S3. LC-ESI-HRMS analysis of the production of the gentamicin C complex and gentamicin-relate intermediates by *Micromonospora echinospora* mutants fed with gentamicin A or G418. Related to Figure 2.

Figure S4. Characterization of purified recombinant GenD2, GenS2, GenN and GenD1. Related to Figures 3 and 4.

Figure S5. LC-ESI-HRMS analysis of GenD2-, GenS2- and GenN-catalyzed modifications on kanamycin B and tobramycin. Related to Figure 3.

Supplemental Experimental Procedures

1. List of oligonucleotide primers used in this study
2. List of bacterial strains and plasmids used in this study
3. Structures of 3''-dehydro-3''-amino-gentamicin A2 and its homologs
4. Purification of gentamicin A2
5. Kinetic study of GenN with kanamycin B
6. Conditions for LC-ESI-MS analyses

Table S1. LC-HRMS analysis of *genD2-genS2-genN-genD1* knock-out mutants

Strain	Gentamicin C complex production (×10^5)					Intermediates production (×10^5				
	C1a	C2b	C2	C2a	C1	A2	A	A2e	X2	DAA2
wild-type	12.6	1.3	59.8	10.0	95.9	2.1	3.4	ND	2.2	ND
ΔgenD2	ND	ND	ND	ND	ND	25.6	ND	30.3	ND	ND
ΔgenS2	ND	ND	ND	ND	ND	47.4	ND	46.5	ND	ND
ΔgenD2ΔgenK	ND	ND	ND	ND	ND	69.1	ND	ND	ND	ND
ΔgenS2ΔgenK	ND	ND	ND	ND	ND	70.6	ND	ND	ND	ND
ΔgenD2ΔgenK::*genK*	ND	ND	ND	ND	ND	27.8	ND	42.3	ND	ND
ΔgenS2ΔgenK::*genK*	ND	ND	ND	ND	ND	27.8	ND	36.8	ND	ND
ΔgenD1	ND	ND	ND	ND	ND	13.7	19.7	5.6	ND	ND
ΔgenN	ND	ND	ND	ND	ND	19.1	ND	18.1	ND	0.7

ND, not detected

Figure S1. In-frame deletion of *genD2*, *genS2*, *genN* and *genD1* in *M. echinospora* ATCC 15835, and complementation of ΔgenD2, ΔgenS2, ΔgenN, ΔgenD1, ΔgenD2ΔgenK and ΔgenS2ΔgenK. Related to Figure 2.

Schematic representation of the in-frame deletions and complementations, and Southern blot confirmations are shown for each mutant. The arrows indicate the expected size of the fragments from the wild-type and mutants chromosomal DNA, respectively.

A. ΔgenD2

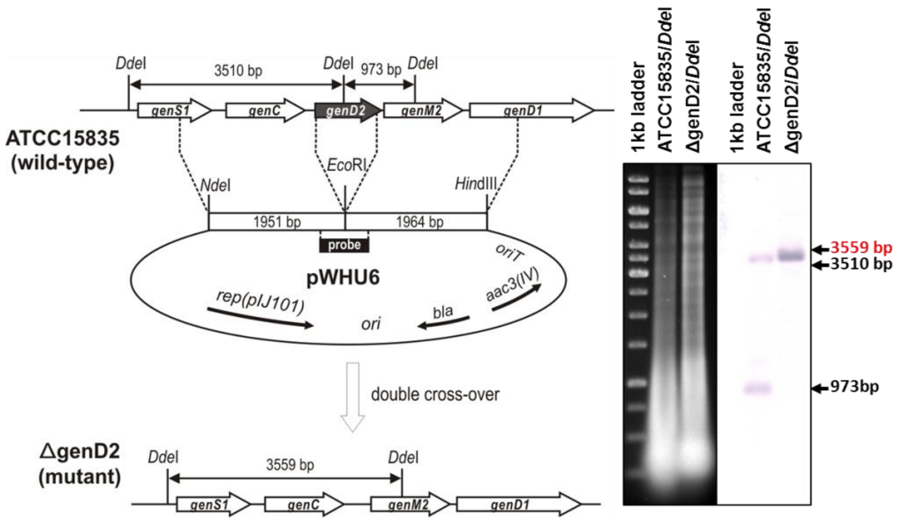

B. ΔgenS2

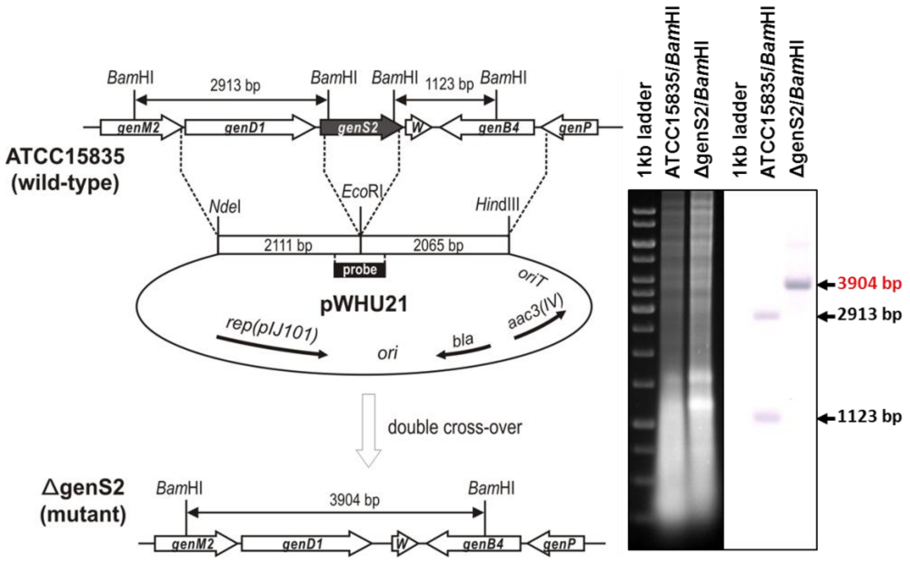

C. ΔgenD2ΔgenK

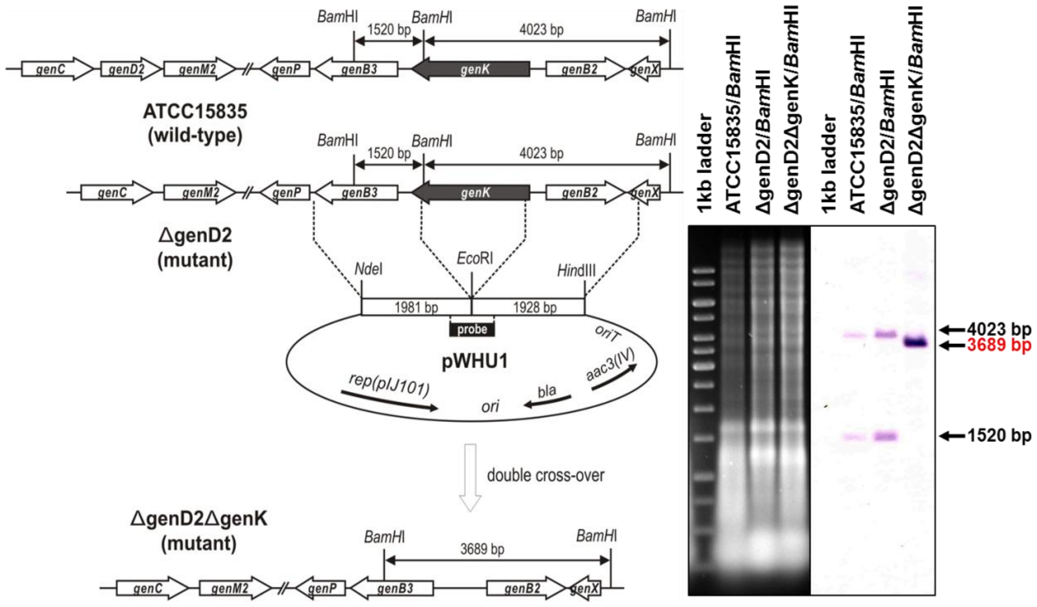

D. ΔgenS2ΔgenK

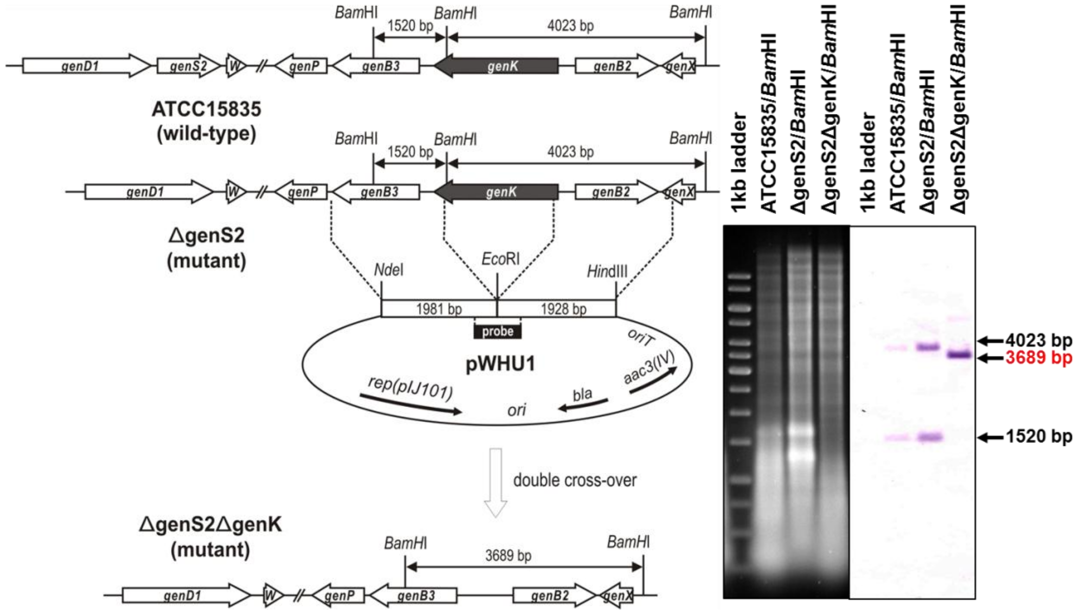

E. ΔgenN

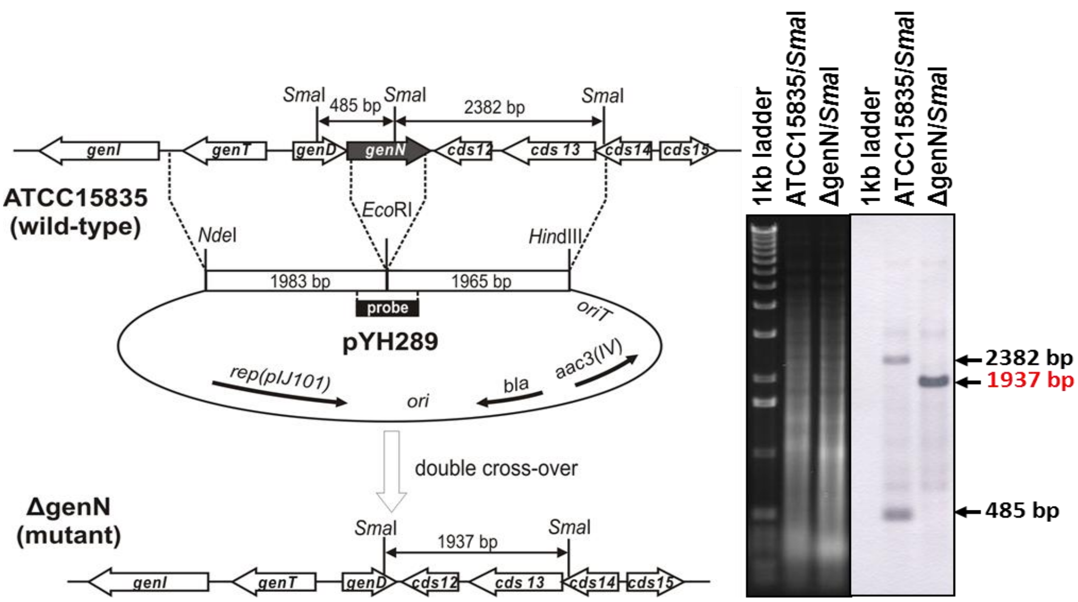

F. ΔgenD1

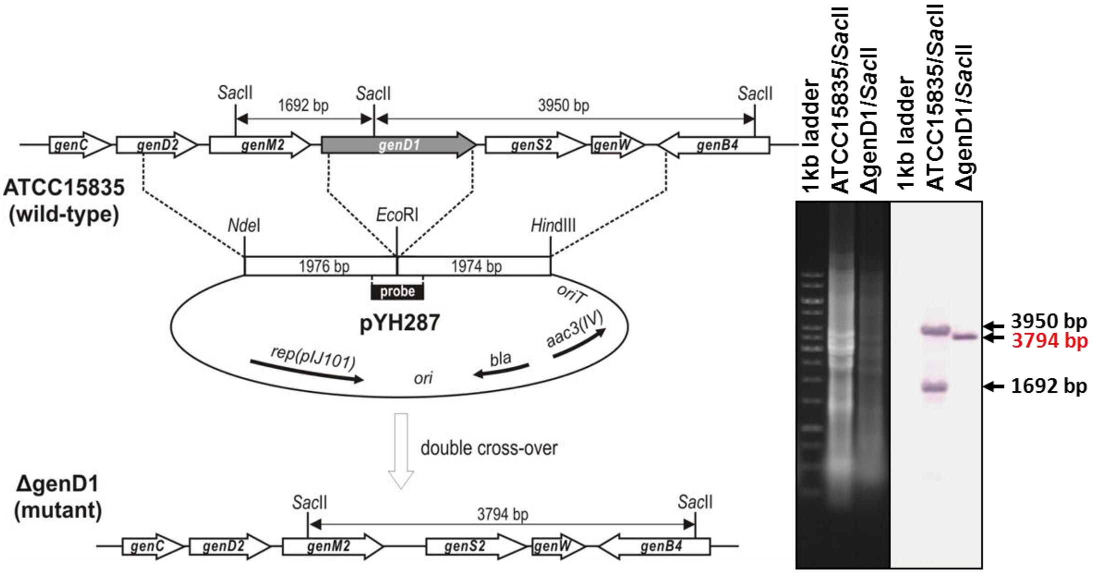

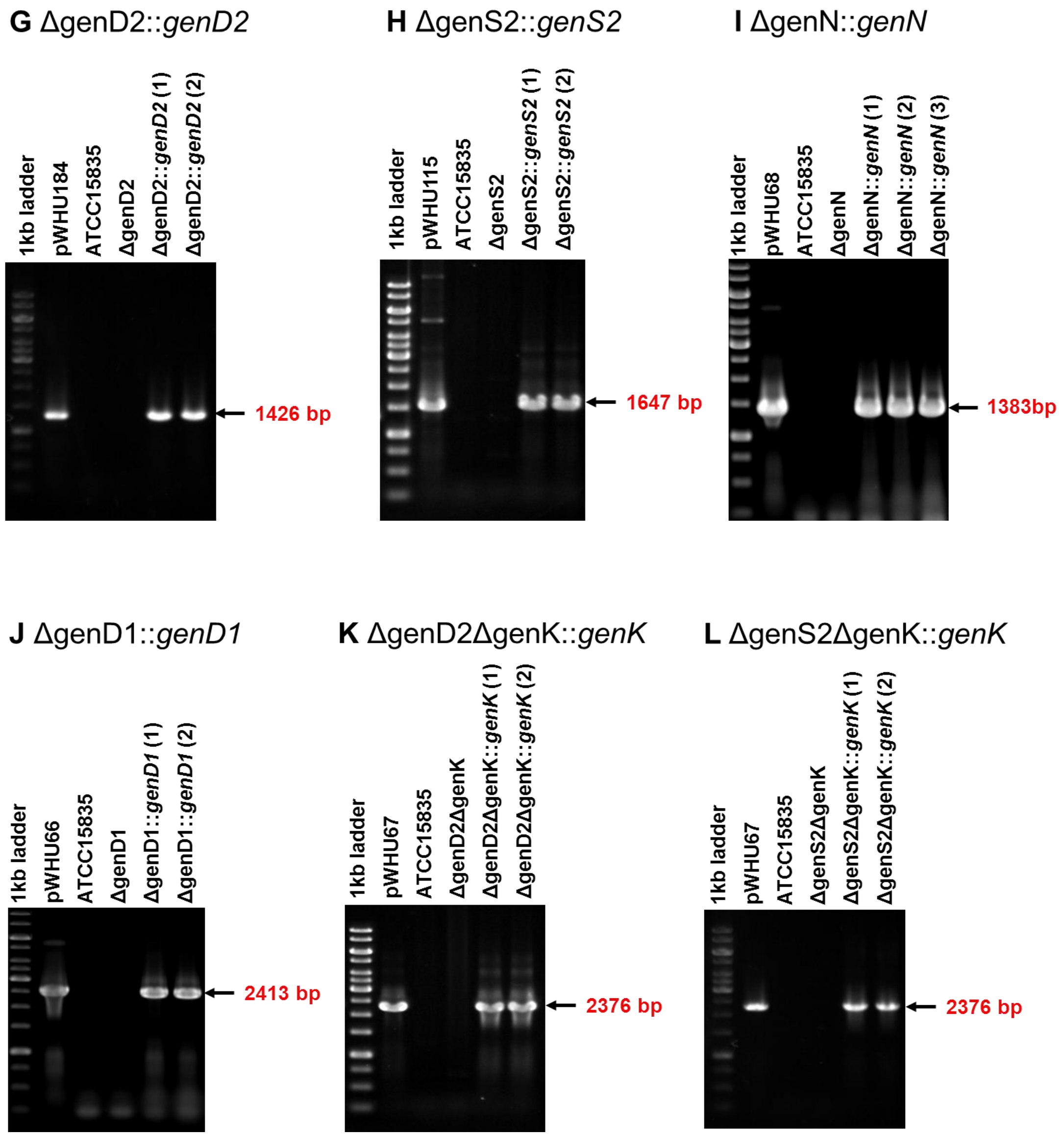
G ΔgenD2::genD2
1kb ladder
pWHU184
ATCC15835
ΔgenD2
ΔgenD2::genD2 (1)
ΔgenD2::genD2 (2)
1426 bp
H ΔgenS2::genS2
1kb ladder
pWHU115
ATCC15835
ΔgenS2
ΔgenS2::genS2 (1)
ΔgenS2::genS2 (2)
1647 bp
I ΔgenN::genN
1kb ladder
pWHU68
ATCC15835
ΔgenN
ΔgenN::genN (1)
ΔgenN::genN (2)
ΔgenN::genN (3)
1383bp
J ΔgenD1::genD1
1kb ladder
pWHU66
ATCC15835
ΔgenD1
ΔgenD1::genD1 (1)
ΔgenD1::genD1 (2)
2413 bp
K ΔgenD2ΔgenK::genK
1kb ladder
pWHU67
ATCC15835
ΔgenD2ΔgenK
ΔgenD2ΔgenK::genK (1)
ΔgenD2ΔgenK::genK (2)
2376 bp
L ΔgenS2ΔgenK::genK
1kb ladder
pWHU67
ATCC15835
ΔgenS2ΔgenK
ΔgenS2ΔgenK::genK (1)
ΔgenS2ΔgenK::genK (2)
2376 bp

Figure S2. MS and MS/MS spectra of gentamicin-related intermediates isolated from mutants of *M. echinospora*. Related to Figure 2.

Selective ion monitoring on (A) [M+H]$^+$(m/z 456.2188) ion of gentamicin A2 (**3**); (B) [M+H]$^+$(m/z 470.2344) ion of gentamicin A2e (**7**); (C) [M+H]$^+$(m/z 455.23478) ion of 3''-dehydro-3''-amino-gentamicin A2 (DAA2, [**9**]); (D) [M+H]$^+$(m/z 469.2504) ion of gentamicin A (**6**); (E) [M+H]$^+$(m/z 483.2661) ion of gentamicin Ae (**10**)

A

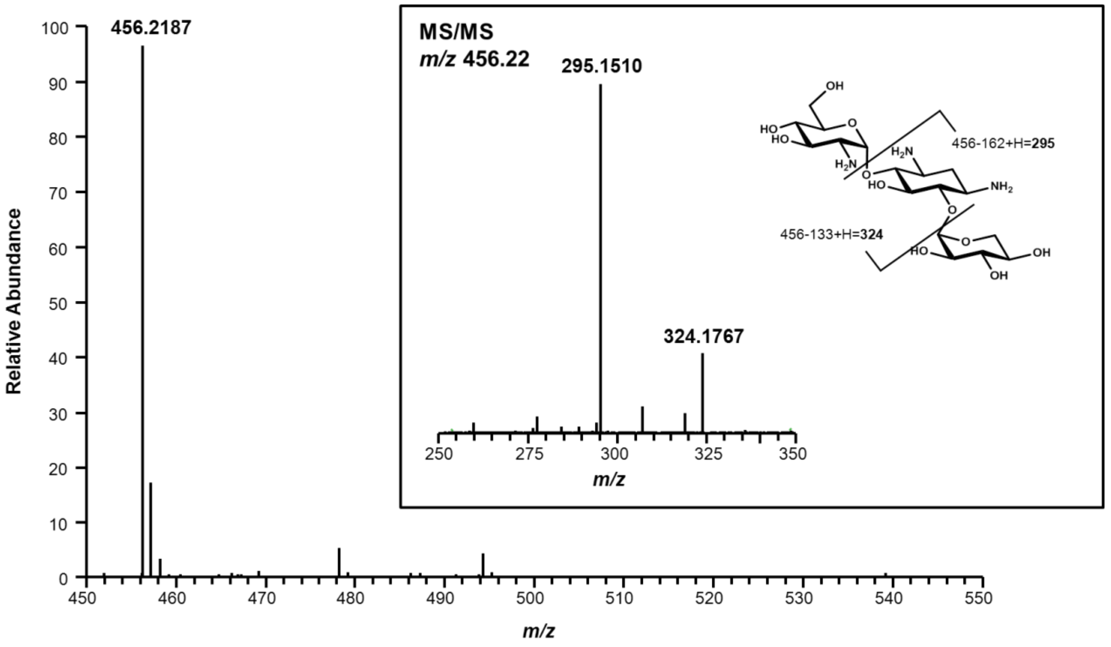

B

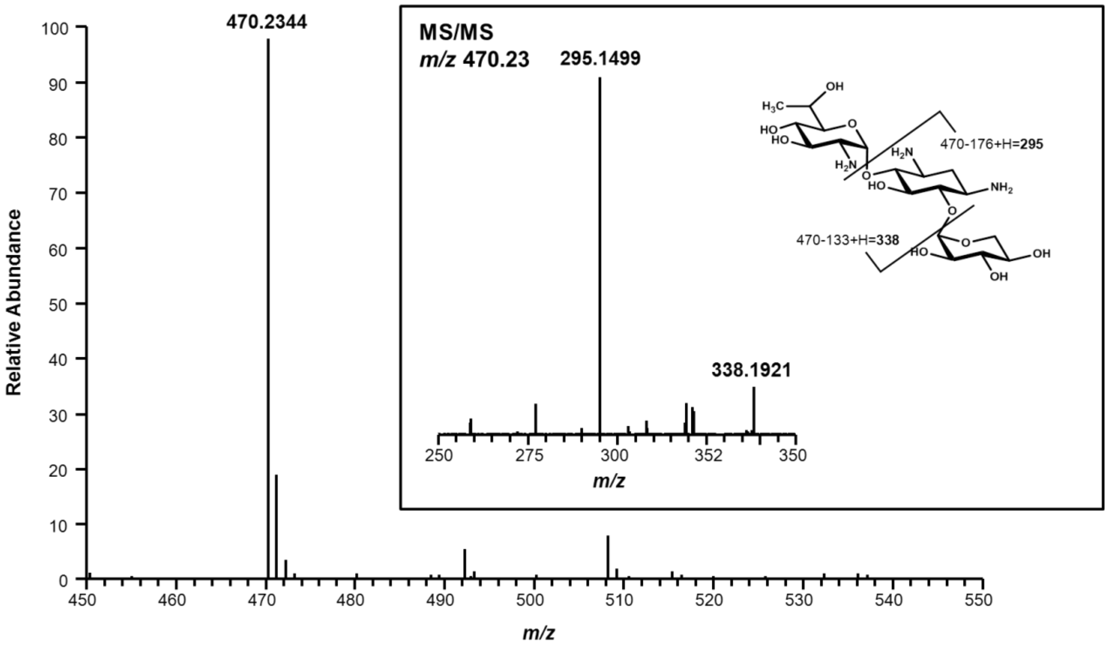

C

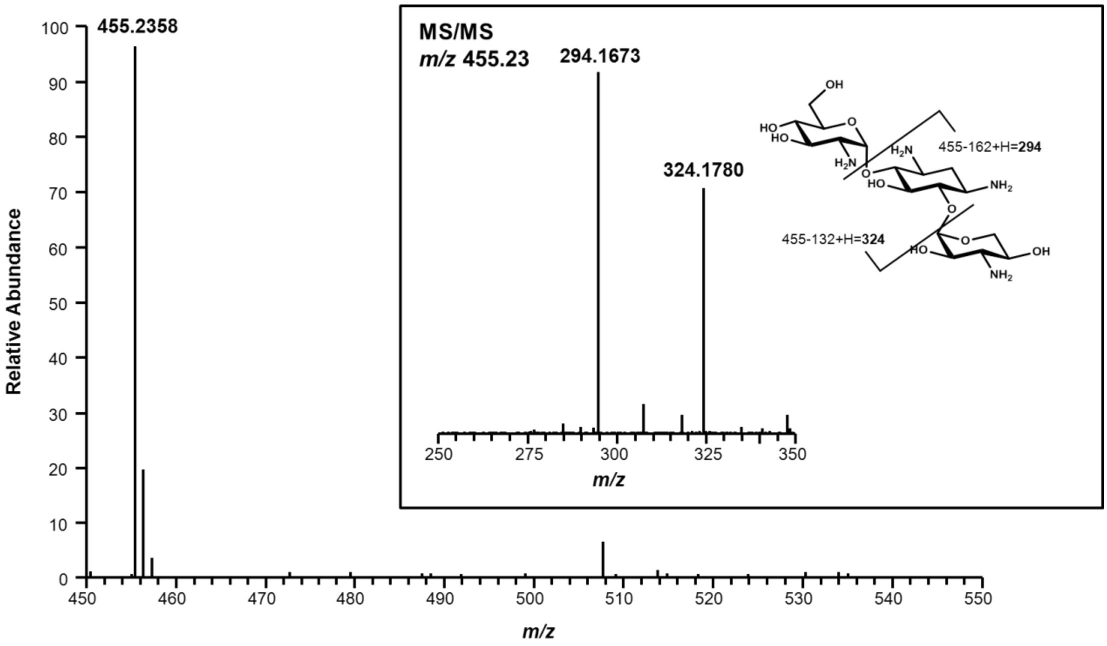
455.2358
Relative Abundance
m/z
MS/MS
m/z 455.23
294.1673
324.1780
455-162+H=294
455-132+H=324

D

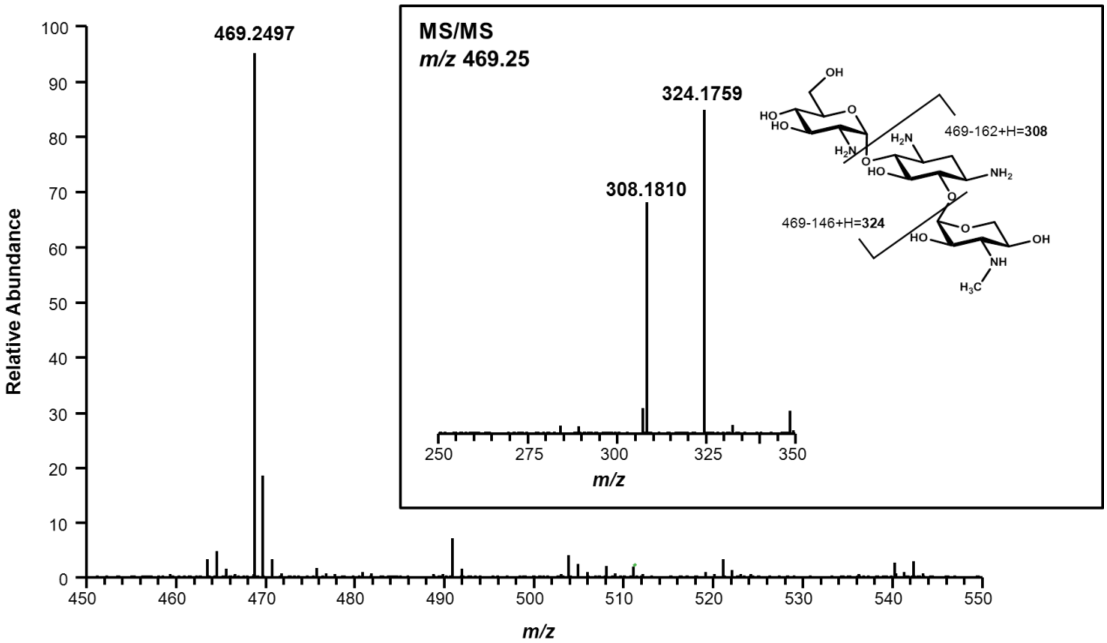
469.2497
Relative Abundance
m/z
MS/MS
m/z 469.25
324.1759
308.1810
469-162+H=308
469-146+H=324

E

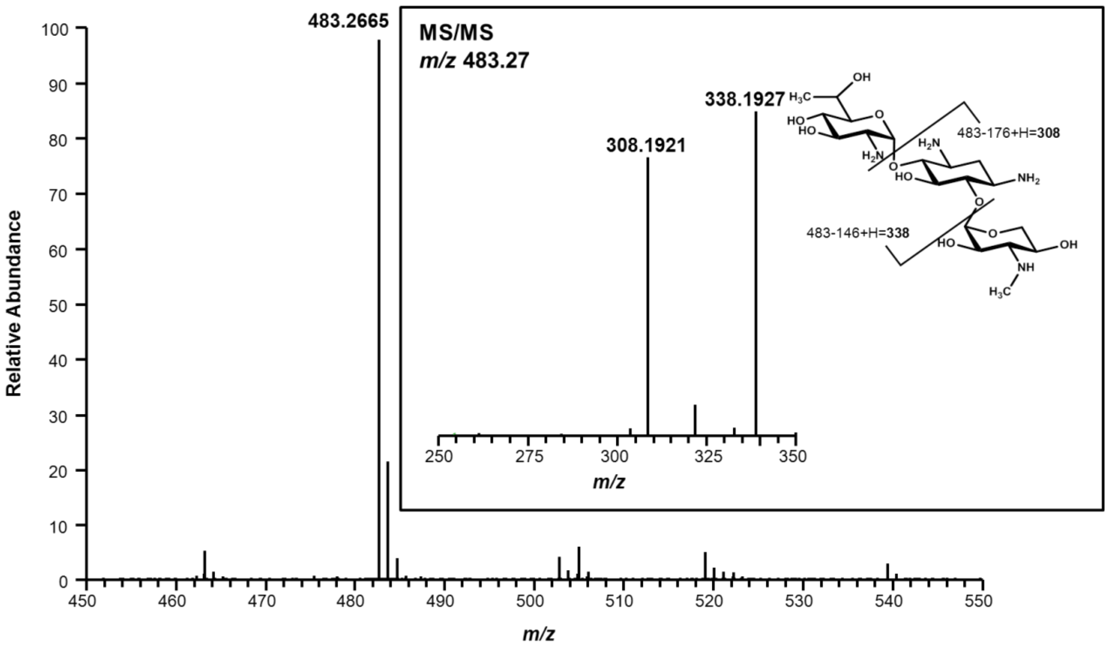
483.2665
Relative Abundance
m/z
MS/MS
m/z 483.27
338.1927
308.1921
483-176+H=308
483-146+H=338

Figure S3. LC-ESI-HRMS analysis of the production of the gentamicin C complex and gentamicin-related metabolites by *Micromonospora echinospora* mutants when fed with gentamicin A or G418. Related to Figure 2.

Total ion current trace of (A) gentamicin standard; and fermentation culture extracts from (B) wild-type; (C) ΔgenD2 mutant fed with gentamicin A; (D) ΔgenS2 mutant fed with gentamicin A; (E) ΔgenN mutant fed with gentamicin A; (F) ΔgenD1 mutant fed with gentamicin A; (G) ΔgenD1 mutant fed with G418 (**5**).

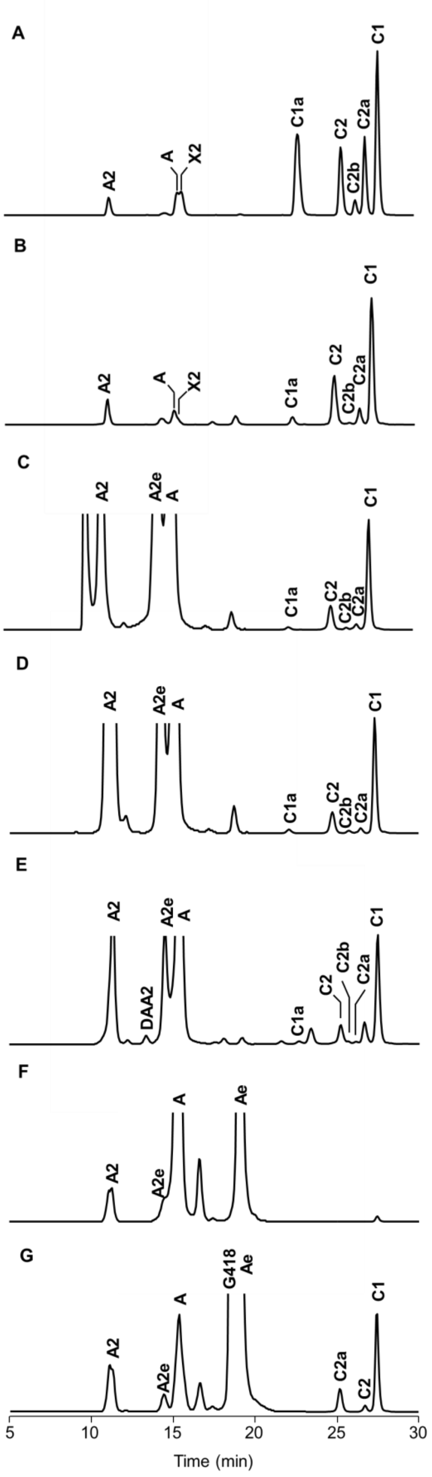

Figure S4. Characterization of purified recombinant GenD2, GenS2, GenN and GenD1. Related to Figure 3 and 4.
(A) SDS-PAGE gel of GenD2, GenS2, GenN and GenD1; (B) UV-visible absorption spectra of GenD2, GenS2 and GenD1.

A

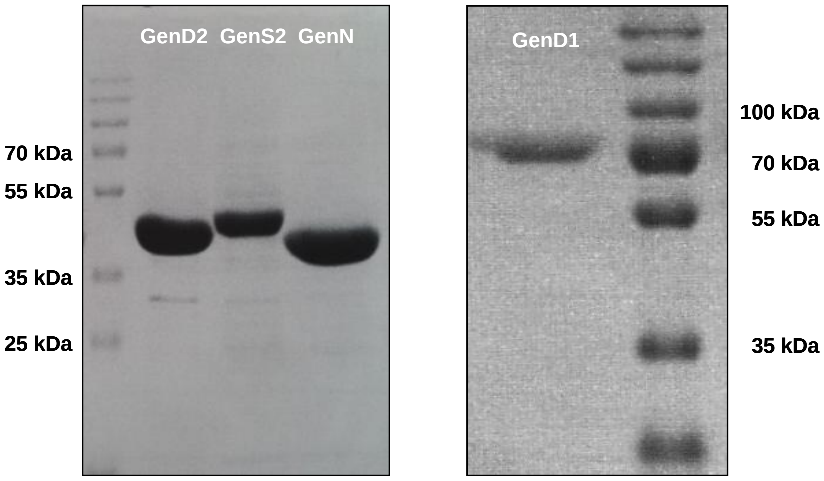

B

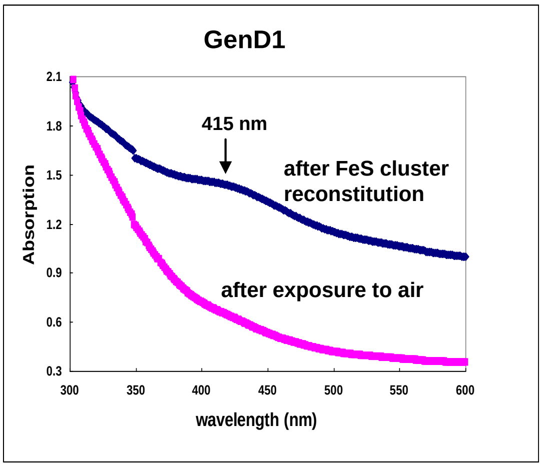

Figure S5. LC-ESI-HRMS analysis of GenD2-, GenS2- and GenN-catalyzed modifications on kanamycin B and tobramycin. Related to Figure 3.
Selective ion monitoring was carried out on
(A) $[M+H]^+$ (m/z 484) and $[M+Na]^+$ (m/z 506) ions of kanamycin B (**11**);
(B) $[M+H]^+$ (m/z 483) and $[M+Na]^+$ (m/z 505) ions of 3''-deamino-3''-oxo-kanamycin B (**13**), the product of GenS2 catalyzed de-amination of **11**;
(C) $[M+H]^+$ (m/z 485) and $[M+Na]^+$ (m/z 507) ions of 3''-deamino-3''-hydroxy-kanamycin B (**15**), the product of GenD2 catalyzed hydrogenation of **13**;
(D) $[M+H]^+$ (m/z 498) and $[M+Na]^+$ (m/z 520) ions of 3''N-methyl-kanamycin B (**17**), the product of GenN catalyzed methylation of **11**.

(E) $[M+H]^+$ (m/z 468) and $[M+Na]^+$ (m/z 490) ions of tobramycin (**12**);
(F) $[M+H]^+$ (m/z 467) and $[M+Na]^+$ (m/z 489) ions of 3''-deamino-3''-oxo-tobramycin (**14**), the product of GenS2 catalyzed de-amination of **12**;
(G) $[M+H]^+$ (m/z 469) and $[M+Na]^+$ (m/z 491) ions of 3''-deamino-3''-hydroxy-tobramycin (**16**), the product of GenD2 catalyzed hydrogenation of **14**;
(H) $[M+H]^+$ (m/z 482) and $[M+Na]^+$ (m/z 504) ions of 3''N-methyl-tobramycin (**18**), the product of GenN catalyzed methylation of **12**.
MS/MS analysis of $[M+H]^+$ (m/z 484), $[M+H]^+$ (m/z 483), $[M+H]^+$ (m/z 485), $[M+H]^+$ (m/z 498) MS^2 analysis of $[M+H]^+$ (m/z 468), $[M+H]^+$, (m/z 467), $[M+H]^+$, (m/z 485) and $[M+H]^+$ (m/z 482) ions are also shown.

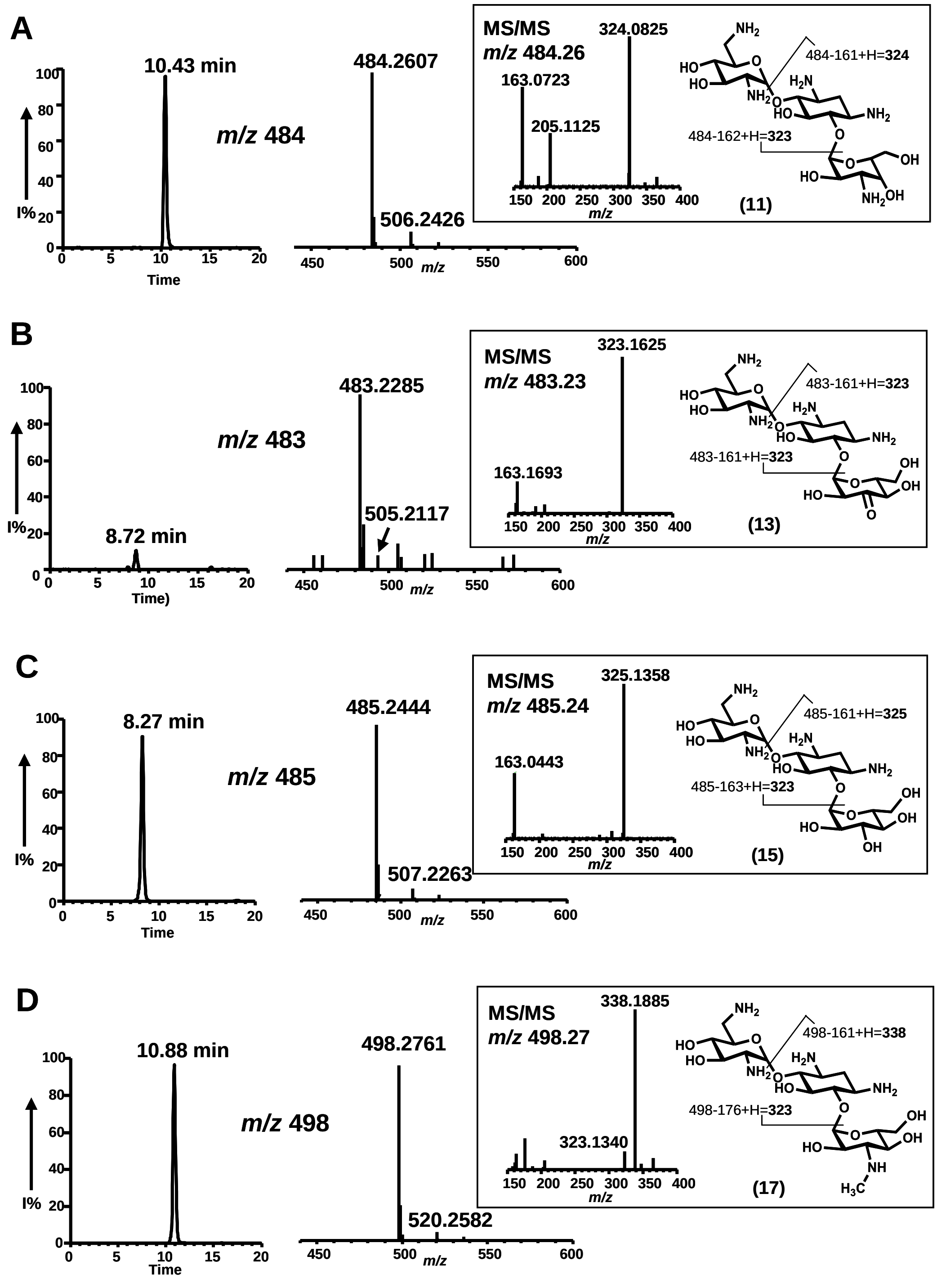

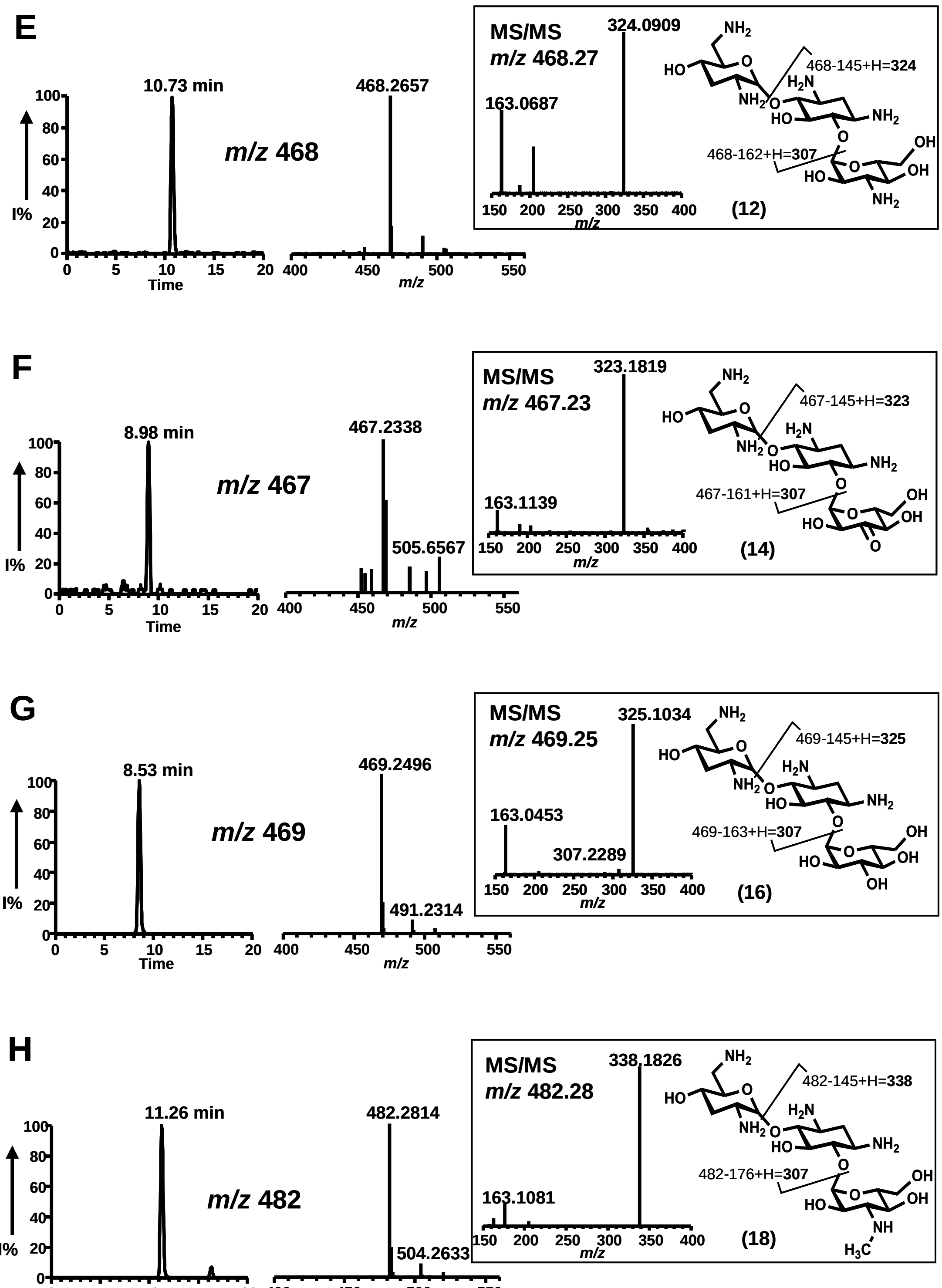
E
10.73 min
468.2657
m/z 468
I%
Time
m/z
MS/MS
m/z 468.27
324.0909
163.0687
468-145+H=324
468-162+H=307
(12)
F
8.98 min
467.2338
505.6567
m/z 467
MS/MS
m/z 467.23
323.1819
163.1139
467-145+H=323
467-161+H=307
(14)
G
8.53 min
469.2496
491.2314
m/z 469
MS/MS
m/z 469.25
325.1034
163.0453
307.2289
469-145+H=325
469-163+H=307
(16)
H
11.26 min
482.2814
504.2633
m/z 482
MS/MS
m/z 482.28
338.1826
163.1081
482-145+H=338
482-176+H=307
(18)

Supplemental Experimental Procedures

1. List of oligonucleotide primers used in this work

Primer	Oligonucleotide sequences (5' to 3')	Restriction Site
genD2-L1	CGC**CATATG**AGAGATGGAACTGGC	*Nde*I
genD2-L2	CCG**GAATTC**CACTCGGGGATC	*Eco*RI
genD2-R1	GTG**GAATTC**AATGCCTGACAACAAG	*Eco*RI
genD2-R2	CAG**AAGCTT**CTCGCCCTCCCG	*Hin*dIII
genD2-CK1	GCTGCGGTTCGACAACAAGC	
genD2-CK2	TTGGACGGGATCGGCAGCAC	
genS2-L1	CGT**CATATG**TGTCGCATTCCCACCG	*Nde*I
genS2-L2	GGC**GAATTC**CTGGTGCATGGTGTTC	*Eco*RI
genS2-R1	GAG**GAATTC**CAGGACATGCTGGATG	*Eco*RI
genS2-R2	CTG**AAGCTT**TACAACATCGGCCAGG	*Hin*dIII
genS2-CK1	TGGAGAACTACTGGGTGAAGCA	
genS2-CK2	TCGACCGTGACCTTGAGGAA	
genD2-a	CACGG**CATATG**CTGCCGATGG	*Nde*I
genD2-b	GGGG**GAATTC**TTGTCAGGCATTCAT	*Eco*RI
genS2-a	CCG**CATATG**ACGCAGAAACTGGCCA	*Nde*I
genS2-b	GCCA**GAATTC**GATCATAGGCTCTTC	*Eco*RI
genK-CK1	CGGGCGAACCTTCGGGATA	
genK-CK2	CCGTCAGCGTTGGCAATAA	
genD1-L1	GGC**CATATG**GCTCGCGGCCG	*Nde*I
genD1-L2	AAG**GAATTC**CGTGAGGGTCGCCACC	*Eco*RI
genD1-R1	CCG**GAATTC**GCCCTCGGGGC	*Eco*RI
genD1-R2	GTG**AAGCTT**GATCGGCCGGACATCG	*Hin*dIII
genD1-CK1	GAAGCTCGCCGATGCCA	
genD1-CK2	CAGGTGAAGGCGGTGGTG	
genD1-a	CGC**CATATG**ACCGTCACTAACAAG	*Nde*I
genD1-b	CCG**GAATTC**TCAGCGGCTACCTGCCCC	*Eco*RI

genN-L1	GCG**<u>CATATG</u>**CTCGTAGACCCAGTTC	*Nde*I
genN-L2	CTG**<u>GAATTC</u>**CGAGCCTCCGACGATC	*Eco*RI
genN-R1	GAC**<u>GAATTC</u>**CTGCGGGGCTGACCCC	*Eco*RI
genN-R2	GAG**<u>AAGCTT</u>**GCCGCCGACTCCGACC	*Hin*dIII
genN-CK1	GGATGGGATGCCAACGACC	
genN-CK2	ACCGCGACGACGATGACG	
genN-a	CGC**<u>CATATG</u>**ATCGTCGGAGGCTCG	*Nde*I
genN-b	CCG**<u>GAATTC</u>**TCAGCCCCGCATGAGCCG	*Eco*RI
pGenD1-For	GGAGTCCT**<u>CATATG</u>**ACCGTCACTAACAAGA	*Nde*I
pGenD1-Rev	5GGCCAGGGCC**<u>GGATCC</u>**GGACGGGGTCGCCA	*Bam*HI
pGenD2-For	GTGGGTGCTG**<u>CATATG</u>**GTTG AGCGCCTGGG	*Nde*I
pGenD2-Rev	CCCAACACGT**<u>GAATTC</u>**CGCCCATCGGGTCG	*Eco*RI
pGenN-For	ACTCTCGGGAGTAG**<u>CATATG</u>**ATCGTCGGAG	*Nde*I
pGenN-Rev	GGTGCGGT**<u>GGATCC</u>**AACCTG TGGCAGGGCC	*Bam*HI
pGenS2-For	GGCAG GTAGCCG**<u>CATATG</u>**ACGCAGAAACTG	*Nde*I
pGenS2-Rev	TCGCCGATCGG**<u>GAATTC</u>**TTCGAGGGATCGG	*Eco*RI

Primer pairs for amplification of left- or right-flanking fragments of a target gene, for PCR/sequencing confirmation, for complementation plasmid construction, and for cloning target genes for over-expression are marked with suffixes –L1/–L2, -R1/-R2, –a/-b, CK1/CK2, or –For/-Rev, respectively.

2. List of bacterial strains and plasmids used in this study

**Strain/Plasmid**	**Characteristics**	**Reference**
E.coil		
DH10B	Host for general cloning	Invitrogen
NovaBlue	Host for general cloning	Novagen
BL21(DE3)	Host for recombinant protein expression	Novagen
ET12567/pUZ8002	Donor strain for conjugation between *E.coli* and *Streptomyces*	MacNeil et al., (1992)
Micromonospora echinospora		
ATCC15835	Gentamicin producing wild-type strain	Weinstein et al., (1963)

ΔgenD2	*genD2* single in-frame deletion mutant	This work
ΔgenS2	*genS2* single in-frame deletion mutant	This work
ΔgenD2ΔgenK	*genD2* and *genK* in-frame deletion mutant	This work
ΔgenS2ΔgenK	*genS2* and *genK* in-frame deletion mutant	This work
ΔgenD2::*genD2*	Self-complementation of *genD2* in ΔgenD2	This work
ΔgenD1::*genD1*	Self-complementation of *genD1* in ΔgenD1	This work
ΔgenS2::*genS2*	Self-complementation of *genS2* in ΔgenS2	This work
ΔgenN::*genN*	Self-complementation of *genN* in ΔgenN	This work
ΔgenD2ΔgenK::*genK*	Self-complementation of *genK* in ΔgenD2ΔgenK	This work
ΔgenS2ΔgenK::*genK*	Self-complementation of *genK* in ΔgenS2ΔgenK	This work
**Plasmid**		
pUC18	Vector for sub-cloning and DNA sequencing	Takara
pYH7	*E. coli-Streptomyces* shuttle vector	Sun et al., (2009)
pWHU1	*genK* in-frame deletion construct	Guo et al. (2014)
pWHU6	*genD2* in-frame deletion construct	This work
pWHU21	*genS2* in-frame deletion construct	This work
pYH287	*genD1* in-frame deletion construct	This work
pYH289	*genN* in-frame deletion construct	This work
pWHU67	*genK* self-complementation construct	Guo et al., (2014)
pWHU115	*genS2* self-complementation construct	This work
pWHU184	*genD2* self-complementation construct	This work
pWHU66	*genD1* self-complementation construct	This work
pWHU68	*genN* self-complementation construct	This work
pET28/genD2	for over-expression of recombinant GenD2	This study
pET28/genS2	for over-expression of recombinant GenS2	This study
pET28/genN	for over-expression of recombinant GenN	This study
pET28/genD1	for over-expression of recombinant GenD1	This study
pDB1282	for over-expression of iron-sulfur cluster (isc) biosynthetic genes	Zheng et al., 1998

3. Structures of 3''-dehydro-3''-amino-gentamicin A2 and its homologs, kanamycin B, tobramycin and sisomicin.

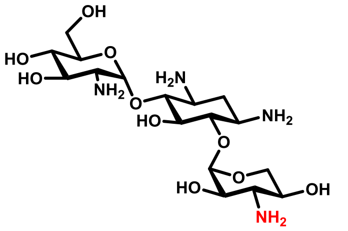

3''-dehydro-3''-amino-gentamicin A2
(DAA2, 9)

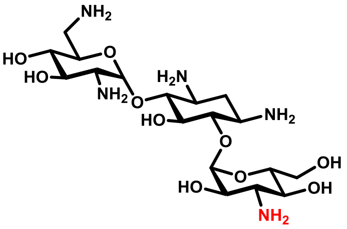

kanamycin B (11)

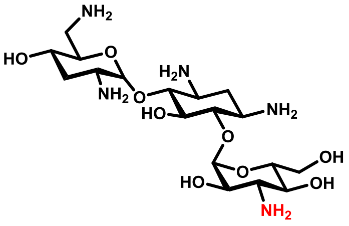

tobramycin (12)

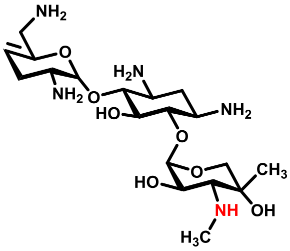

sisomicin (19)

4. Purification of gentamicin A2

Purification of gentamicin A2 (**3**) from extracts of the ΔgenS2ΔgenK mutant fermentation broth after cation exchange (see Experimental Procedures) was performed on a Waters 2535 semi-preparative HPLC system using a ZORBAX SB-C18 (9.4 × 250 mm, 5μ, Agilent) semi-preparative column, with as mobile phase (A) 0.2% TFA in water adjusted to pH 2 with NH_4OH and (B) acetonitrile. Elution of gentamicin A2 was carried out isocratically at 95% A for 15 min at a flow rate of 4 ml/min. The column eluate was directed to an Evaporative Light Scattering Detector(ELSD, Alltech 3300) with 2ml/min splitting, and the rest of the eluate was collected in 1 ml fractions. The atomization temperature of ELSD was 55°C and the drift tube temperature was 48 °C. Fractions containing gentamicin A2 were pooled and further confirmed by LC-ESI-HRMS.

5. Kinetic study of GenN with kanamycin B

8.5 nM GenN was incubated in 500 μl Tris-HCl (50 mM, pH 7.5) buffer at 30°C with kanamycin B (**11**) at concentrations of 30, 40, 50, 60 and 70 μM in the presence of 250 μM SAM. An 80 μl aliquot of each reaction was taken out after 1, 2, 3, 4, 5 and 6 min incubation and mixed immediately with an equal volume of ice cold chloroform to precipitate the enzyme. The reactions were analyzed by LC-ESI-MS. The product 3''N-methylkanamycin B (**17**) was quantified

based on the peak integration of the selected ion. Data points are the mean value of three replicates.

6. Conditions for LC-ESI-MS analyses

LC-ESI-HRMS analysis of gentamicin-related metabolites and in vitro assays were performed on a Thermo LTQ-Orbitrap XL instrument using a Luna C18 column (250 mm × 4.6 mm, 5 µ, Phenomenex) with a flow rate of 0.4 ml/min. LC-MS analysis of proteins was carried out on a ThermoFinnigan LCQ fitted with an ESI source connected to an Agilent HP 1100 HPLC system using a Nucleosil C4 column (250 mm × 2 mm, 5 µ, Macherey-Nagel). The mobile phase and gradients used for HPLC are as follows:

HPLC gradient used for analysis of gentamicin-related metabolites

Mobile phase A: 0.2% TFA in water; Mobile phase B: acetonitrile. 2% B to 14% B over 18 min, then to 90 % B within 1 min, maintained at 90% B for 5 min, returned to 2% B over 1 min and maintained at 2% B for a further 5 min. The injection volume was 5 µl for each gentamicin standard (500 µg ml^{-1}) and 20 µl for each sample. Each cultivation and analysis was performed in triplicate.

HPLC gradient for analysis of aminoglycosides in enzymatic assays

Mobile phase A: 0.2% TFA in water; Mobile phase B: 0.1% TFA in acetonitrile. 2%B to 8%B over 9 min then to 90%B over 1 min, maintained at 90%B for 4 min, returned to 2%B over 1 min and then maintained at 2%B for a further 5 min. flow rate 0.6 ml/min.

HPLC gradient for analysis of 5'-deoxydenosine in enzymatic assays

Mobile phase A: 0.1% TFA in water; Mobile phase B: 0.1% TFA in acetonitrile. 0%B to 20% B over 30 min. 0.6 ml/min.

HPLC gradient for analysis of proteins

Mobile phase A: 0.1% TFA in water; Mobile phase B: 0.1% TFA in acetonitrile. 35% B to 45 % B over 5 min, increasing gradient of B to 75% over 20 min, gradient to 95% within 2 min, maintained at 95% B for 7 min, followed by a gradient from 95% B to 35%B within 3 min;

References

1. Kim J.Y., Suh J.W., Kang S.H., Phan T.H., Park S.H., Kwon H.J.(2008). Gene inactivation study of gntE reveals its role in the first step of pseudotrisaccharide modifications in gentamicin biosynthesis. Biochem. Biophys. Res. Commun. *372*, 730-734.

2. MacNeil, D.J., Occi, J.L., Gewain, K.M., MacNeil, T., Gibbons, P.H., Ruby, C.L., and Danis, S.J. (1992). Complex organization of the *Streptomyces avermitilis* genes encoding the avermectin polyketide synthase. Gene *115*,

119-125.

3. Weinstein, M.J., Luedemann, G.M., Oden, E.M., Wagman, G.H., Rosselet, J.P., Marquez, J.A., Coniglio, C.T., Charney, W., Herzog, H.L., and Black, J (1963). Gentamicin, a new antibiotic complex from *Micromonospora*. J. Med. Chem. *6*, 463-464.

4. Sun, Y., He, X., Liang, J., Zhou X., and Deng, Z. (2009). Analysis of functions in plasmid pHZ1358 influencing its genetic and structural stability in *Streptomyces lividans* 1326. Appl. Microbiol. Biotechnol. *82*, 303-310.

5. Guo, J., Huang, F., Huang, C., Duan, X., Jian, X., Leeper, F., Deng, Z., and Leadlay, P.F., Sun, Y. (2014). Specificity and promiscuity at the branch point in gentamicin biosynthesis. Chem Biol. *21*, 608-618.
